# Surfactin Stimulated by Pectin Molecular Patterns and Root Exudates Acts as a Key Driver of the *Bacillus*-Plant Mutualistic Interaction

**DOI:** 10.1128/mBio.01774-21

**Published:** 2021-11-02

**Authors:** Grégory Hoff, Anthony Arguelles Arias, Farah Boubsi, Jelena Pršić, Thibault Meyer, Heba M. M. Ibrahim, Sébastien Steels, Patricio Luzuriaga, Aurélien Legras, Laurent Franzil, Michelle Lequart-Pillon, Catherine Rayon, Victoria Osorio, Edwin de Pauw, Yannick Lara, Estelle Deboever, Barbara de Coninck, Philippe Jacques, Magali Deleu, Emmanuel Petit, Olivier Van Wuytswinkel, Marc Ongena

**Affiliations:** a Microbial Processes and Interactions, TERRA Teaching and Research Center, BioEcoAgro, Joint Research Unit/UMR transfrontalière 1158, University of Liège ‐Gembloux Agro‐Bio Tech, Gembloux, Belgium; b Ecology and Biodiversity, Department of Biology, Utrecht University, Utrecht, The Netherlands; c UMR Ecologie Microbienne, F-69622, University of Lyon, Université Claude Bernard Lyon 1, CNRS, INRAE, VetAgro Sup, Villeurbanne, France; d Division of Plant Biotechnics, Department of Biosystems, Faculty of Bioscience Engineering, KU Leuven, Leuven, Belgium; e Unité Biologie des Plantes et Innovation, BioEcoAgro, Joint Research Unit/UMR Transfrontalière 1158, Université de Picardie Jules Verne, UFR des Sciences, Amiens, France; f Mass Spectrometry Laboratory, MolSys Research Unit, Department of Chemistry, University of Liège, Liège, Belgium; g Astrobiology, UR-ASTROBIOLOGY, Geology Department, University of Liège, Liège, Belgium; h Molecular Biophysics at Interfaces Laboratory, Gembloux Agro-Bio Tech, University of Liège, Gembloux, Belgium; University of Nebraska-Lincoln

**Keywords:** lipopeptides, plant cell wall polymers, plant immunity, molecular crosstalk, plant-microbe interactions

## Abstract

Bacillus velezensis is considered as a model species belonging to the so-called Bacillus subtilis complex that evolved typically to dwell in the soil rhizosphere niche and establish an intimate association with plant roots. This bacterium provides protection to its natural host against diseases and represents one of the most promising biocontrol agents. However, the molecular basis of the cross talk that this bacterium establishes with its natural host has been poorly investigated. We show here that these plant-associated bacteria have evolved a polymer-sensing system to perceive their host and that, in response, they increase the production of the surfactin-type lipopeptide. Furthermore, we demonstrate that surfactin synthesis is favored upon growth on root exudates and that this lipopeptide is a key component used by the bacterium to optimize biofilm formation, motility, and early root colonization. In this specific nutritional context, the bacterium also modulates qualitatively the pattern of surfactin homologues coproduced *in planta* and forms mainly variants that are the most active at triggering plant immunity. Surfactin represents a shared good as it reinforces the defensive capacity of the host.

## INTRODUCTION

Soil is among the richest ecosystems in terms of microbial diversity, but only a subset of these microbes has evolved to efficiently establish in the competitive and nutrient-enriched rhizosphere layer surrounding plant roots (1). The rhizosphere includes plant beneficial bacteria dwelling on the rhizoplane as multicellular biofilm communities that feed on exuded carbohydrates (2, 3) and, in turn, contribute to host fitness via growth stimulation and protection against phytopathogens (4, 5). This biocontrol activity is mediated via competition for nutrients and space, direct growth inhibition of the pathogenic (micro)organisms, and more indirectly by stimulating the host defensive capacity in an immunization-like process which leads to induced systemic resistance (ISR) (6, 7). This ISR mechanism results in enhanced defense lines and reduced disease symptoms upon perception of plant beneficial microbes (6, 8).

From an ecological viewpoint, rhizosphere establishment and persistence of these beneficial bacteria rely on various traits, but efficient root colonization and high competitiveness toward the surrounding microbiological network are pivotal. It is hypothesized that the potential to produce a wide range of chemically diverse and bioactive secondary metabolites (BSMs) acting as signals and/or antimicrobials is a common key feature of these beneficial bacteria (5, 9, 10). Members of Bacillus velezensis are considered archetypes of plant-associated beneficial bacilli and are among the most prolific BSM producers with more than 12% of their genome devoted to the synthesis of compounds contributing to both ecological competence and biocontrol activity (11–15). Among their BSM arsenal, the cyclic lipopeptide surfactin is synthesized nonribosomally by a multimodular megaenzyme machinery (encoded by the *srfA* operon) and is formed as a mix of naturally coproduced homologues with fatty acid chains of various lengths. This multifunctional compound is of particular interest because it retains important roles in key developmental processes, such as bacterial motility, biofilm formation, and root colonization (16–18), but also because it represents the best described *Bacillus* triggers for plant immunity (6, 8). The potential of surfactin to stimulate ISR has been demonstrated on various plants, including *Solanaceae* like tobacco and tomato on which it acts as a main if not sole elicitor formed by Bacillus subtilis and *B. velezensis* (10, 19). In support to its key role in the interaction with the host plant, we also reported previously that surfactin is formed promptly in the course of early colonization and that its production is stimulated upon sensing root tissues (20).

However, in contrast to the well-studied interactions between plants and microbial pathogens or nitrogen-fixing bacteria (21), relatively little is known about the molecular basis of cooperative interactions between plants and beneficial bacteria, such as *B. velezensis* (11, 20, 22). More specifically, how and to what extent the expression of key bacterial BSMs may be modulated by plant factors are poorly understood. A better knowledge is critical not only for providing new insights in rhizosphere chemical ecology but also for optimizing the use of these species as biocontrol agents, which still suffer from insufficient efficacy in practice (23). Here, we investigated the molecular interaction driving the early steps of partnership establishment between plant roots and *B. velezensis*. We show that cell wall pectin acts in synergy with soluble root exudates as plant host cues perceived by *B. velezensis*. In response, the bacterium stimulates the production of specific surfactin variants as key components of its secretome to further improve the fitness of both partners, i.e., early root colonization and thus rhizosphere competence of the bacterium and priming of immunity in the host plant.

## RESULTS

### Pectin fragments of a high polymerization degree act as host cues triggering surfactin production.

**FIG 1:**
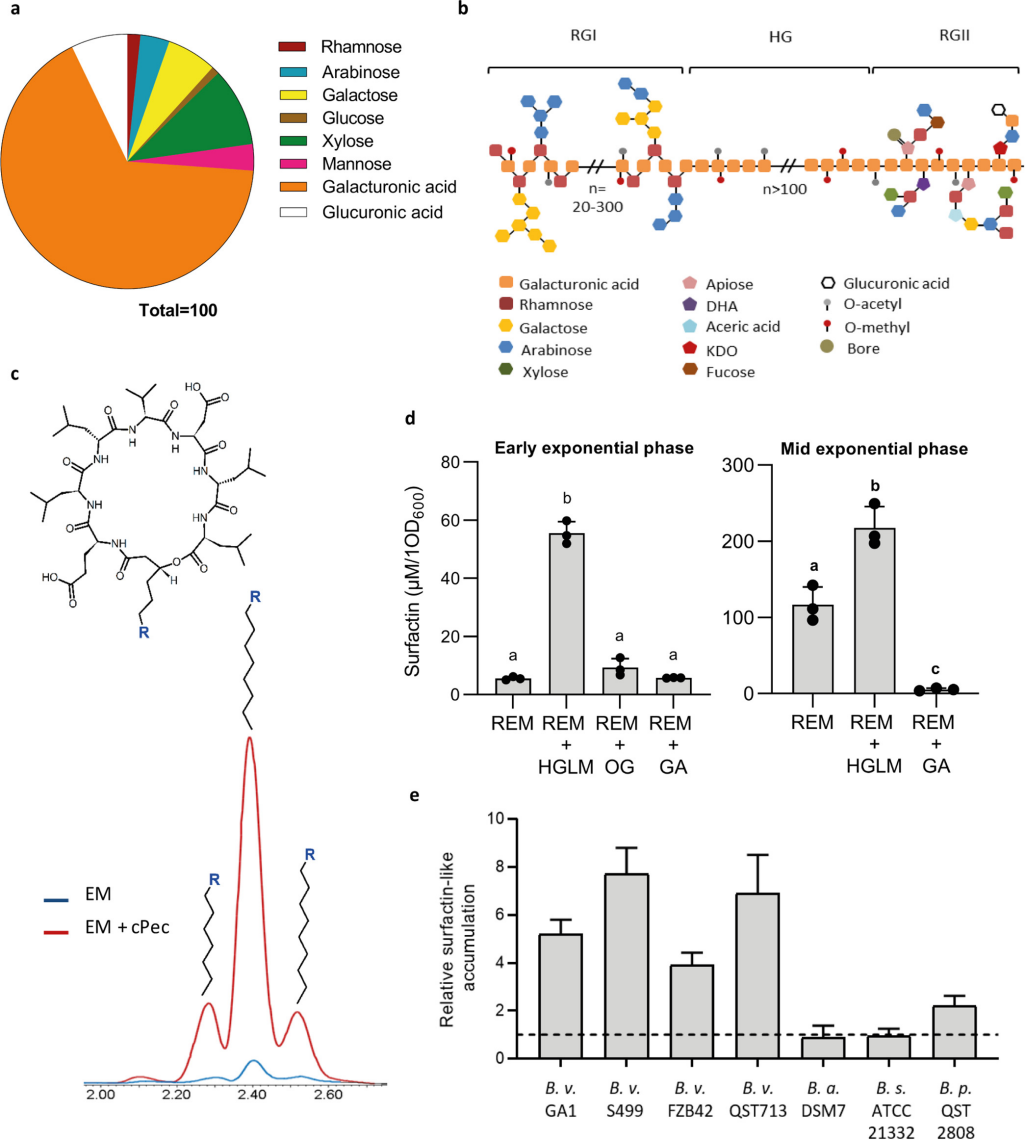
Impact of pectin on early surfactin production. (a) Sugar composition analysis of crude pectin (cPec) extracted from tobacco roots. Composition is expressed as molar ratio percentage (molar %) for each fraction. Galacturonic acid (orange) constituting the pectin backbone (b for schematization) is the main sugar of the cPec fraction. Other minor sugars (e.g., rhamnose, galactose, and arabinose) are found typically in the pectin side chains (24). (b) Schematization of pectin structure. Homogalacturonan (HG) contains an assembly of at least 100 galacturonic acid (GalA) residues that can be acetyl- or methyl-esterified. Rhamnogalacturonan I (RGI) is constituted by a succession of GalA-Rha dimers, with each one containing an alternance of rhamnosyl and galacturonic acid units. The Rha unit can be branched with variable neutral sugar side chains, including essentially galactosyl and/or arabinosyl units. The rhamnogalacturonan II (RGII) structure is well conserved within the HG polymer. RGII englobes 9 GalA units substituted by four side chains with complex sugars, including apiose, dihydroxyacetone (DHA), aceric acid, and ketodeoxyoctonic acid (KDO); neutral sugars like, rhamnose, galactose, arabinose, xylose, and fucose; or also organic acids, such as galacturonic and glucuronic acid. RGII can also complex with bore, allowing a cross link between two HG molecules. (c) Surfactin (cyclic structure represented at top) production in a root exudates mimicking (REM) medium at an early growth phase (OD_600_, 0.2) with (red chromatogram) or without (blue chromatogram) crude pectin extract added to the GA1 cultures. The main peak represents C_15_ surfactin, whereas the minor left and right peaks represent C_14_ and C_16_ surfactins, respectively. (d) Surfactin accumulation in the early- (left panel, OD_600_ of 0.2) and mid- (right panel, OD_600_ of 0.35) exponential growth phase of GA1 cultures in REM supplemented with different sized pectin fragments, as follows: homogalacturonan low methylated (HGLM), DP of >150; oligogalacturonides (OG), DP of 15; galacturonic acid (GA), DP of 1. Means ± SE from three biological replicates of one experiment are shown. Significant difference between each condition is indicated by different letters, *P* < 0.01. (e) Comparison of surfactin induction level by HGLM in the early-exponential growth phase for different *Bacillus* species, as follows: Bacillus velezensis (*B. v*), Bacillus amyloliquefaciens (*B. a*), Bacillus subtilis (*B. s*), and Bacillus pumilus (*B. p*). For each strain tested, surfactin accumulation was normalized with the control condition without HGLM represented by the black dotted line. Means ± SE from three biological replicates are shown.

We described previously that early production of surfactin, as a mix of naturally coproduced homologues with fatty acid chains of various lengths, is stimulated in contact with root tissues and several plant cell wall-associated polymers (PCWPs) (20). In this work, we further investigated this phenomenon focusing on the impact of pectin, as it represents complex sugar polymers typically found in the plant primary cell wall and particularly abundant in the middle lamella layer (24). We first tested the effect of crude pectin extracted from tobacco root PCWPs (referred as cPec) (Fig. 1a and b for composition and related structure). An 8-fold increase of surfactin production was detected at the early exponential growth phase (optical density at 600 nm [OD_600_], 0.2 to 0.25) in *B. velezensis* GA1 liquid cultures supplemented with cPec compared with an unsupplemented culture (Fig. 1c and d). Surfactin production was also 10 times enhanced upon addition at the same concentration of pure commercially available homogalacturonan (HG) with a high degree of polymerization (DP) (see Fig. S1a and b in the supplemental material) but a low level of methyl-esterification (HGLM) according to the manufacturer (Fig. 1d). HGLM was tested as the most abundant pectic polysaccharide constituent, which represents 65% of the crude primary cell wall pectin (24). Production of this lipopeptide was also enhanced to a similar level upon addition of highly methylated HG (HGHM), showing that the degree of methyl-esterification of the polymer is not a major trait influencing perception by the bacterium (see Fig. S2 in the supplemental material). Altogether, this information supports a key role of the pectin backbone as a plant molecular pattern that is sensed by the bacterium to stimulate surfactin synthesis.

10.1128/mBio.01774-21.1 FIG S1 (a and b) Size-exclusion chromatography combined with multiangle laser light scattering (SEC-Malls) characterization of high and low methylated homogalacturonan. (a) SEC-Malls profile of high (blue) and low (green) methylated homogalacturonan. Light curves represent the molecular weight distribution, and dark curves represent the refractive index (RI) signal. (b) SEC-Malls results. Mn, number average molecular weight; MW, weight-average molecular weight; Mw/Mn, polydispersity values. (c) Characterization of oligogalacturonide (OG) polymerization degree by hydrophilic interaction liquid chromatography-quadrupole time of flight (HILIC-QTOF). Download FIG S1, TIF file, 0.4 MB. Copyright © 2021 Hoff et al. 2021 Hoff et al. https://creativecommons.org/licenses/by/4.0/ This content is distributed under the terms of the Creative Commons Attribution 4.0 International license.

10.1128/mBio.01774-21.2 FIG S2 Relative surfactin accumulation by GA1 cells at early growth phase (OD_600_, 0.2) after addition of low (HGLM) or high (HGHM) methyl-esterified HG. Means ± SE from three biological replicates of one experiment are shown; ns, nonsignificant. Download FIG S2, TIF file, 0.02 MB. Copyright © 2021 Hoff et al. 2021 Hoff et al. https://creativecommons.org/licenses/by/4.0/ This content is distributed under the terms of the Creative Commons Attribution 4.0 International license.

Interestingly, by screening the CAZy database (25) for genes encoding carbohydrate-active enzymes potentially involved in PCWP degradation by *B. velezensis*, two putative pectate/pectin lyase-encoding genes were detected. These two genes, referred as *pelA* and *pelB* (locus tags GL331_08735 and GL331_04125 in *B. velezensis* GA1, respectively), are highly conserved among all sequenced *Bacillus* genomes that belong to the “operational group Bacillus amyloliquefaciens” (see Table S1 in the supplemental material) (26). *pelA* and *pelB* are expressed readily in GA1 *in vitro*, and the corresponding enzymes efficiently convert HGLM into unsaturated oligogalacturonides with consistent activity occurring at the beginning of stationary phase (see Fig. S3 in the supplemental material). However, the bacterial perception of oligomers with a lower polymerization degree than HGLM is not obvious since oligogalacturonides (OGs) did not stimulate surfactin biosynthesis (Fig. 1d; Fig. S1c for OG characterization). Supplementation with galacturonic acid (GA) led to a reduction of surfactin production at mid-exponential phase (OD_600_, 0.35) (Fig. 1d). Surfactin production is thus specifically boosted upon sensing long degree of polymerization (DP) polymers but is somehow inhibited in the presence of GA constituting the pectin backbone. Such HGLM-driven surfactin stimulation also occurs in other *B. velezensis* isolates tested (FZB42, QST713, and S499) and to a lower extent in Bacillus pumilus QST 2808. It does not occur in the non-rhizosphere-dwelling isolates *B. amyloliquefaciens* DSM 7 or B. subtilis ATCC 21332 (Fig. 1e), suggesting that this trait may be specific to bacilli with a plant-associated lifestyle.

10.1128/mBio.01774-21.3 FIG S3 Characterization of *pel* expression and pectate lyase activity in GA1. (a) Evolution of *pelA* (gray) and *pelB* (red) expression pattern (*n* = 3). For each time point, means ± SE from three biological replicates of one experiment are shown. (b) Evolution of global pectate lyase activity in a 48-h time course experiment. The box plots encompass the 1st and 3rd quartiles, the whiskers extend to the minimum and maximum points, and the midline indicates the median (*n* = 6 biological replicates of 2 experiments). Significant differences are indicated by different letters (*n* = 6). Download FIG S3, TIF file, 0.08 MB. Copyright © 2021 Hoff et al. 2021 Hoff et al. https://creativecommons.org/licenses/by/4.0/ This content is distributed under the terms of the Creative Commons Attribution 4.0 International license.

10.1128/mBio.01774-21.8 TABLE S1 Conservation of the pectate lyase (*pel*) genes in the “operational group *B. amyloliquefaciens*”. Download Table S1, DOCX file, 0.03 MB. Copyright © 2021 Hoff et al. 2021 Hoff et al. https://creativecommons.org/licenses/by/4.0/ This content is distributed under the terms of the Creative Commons Attribution 4.0 International license.

### The root nutritional context favors early surfactin production.

**FIG 2:**
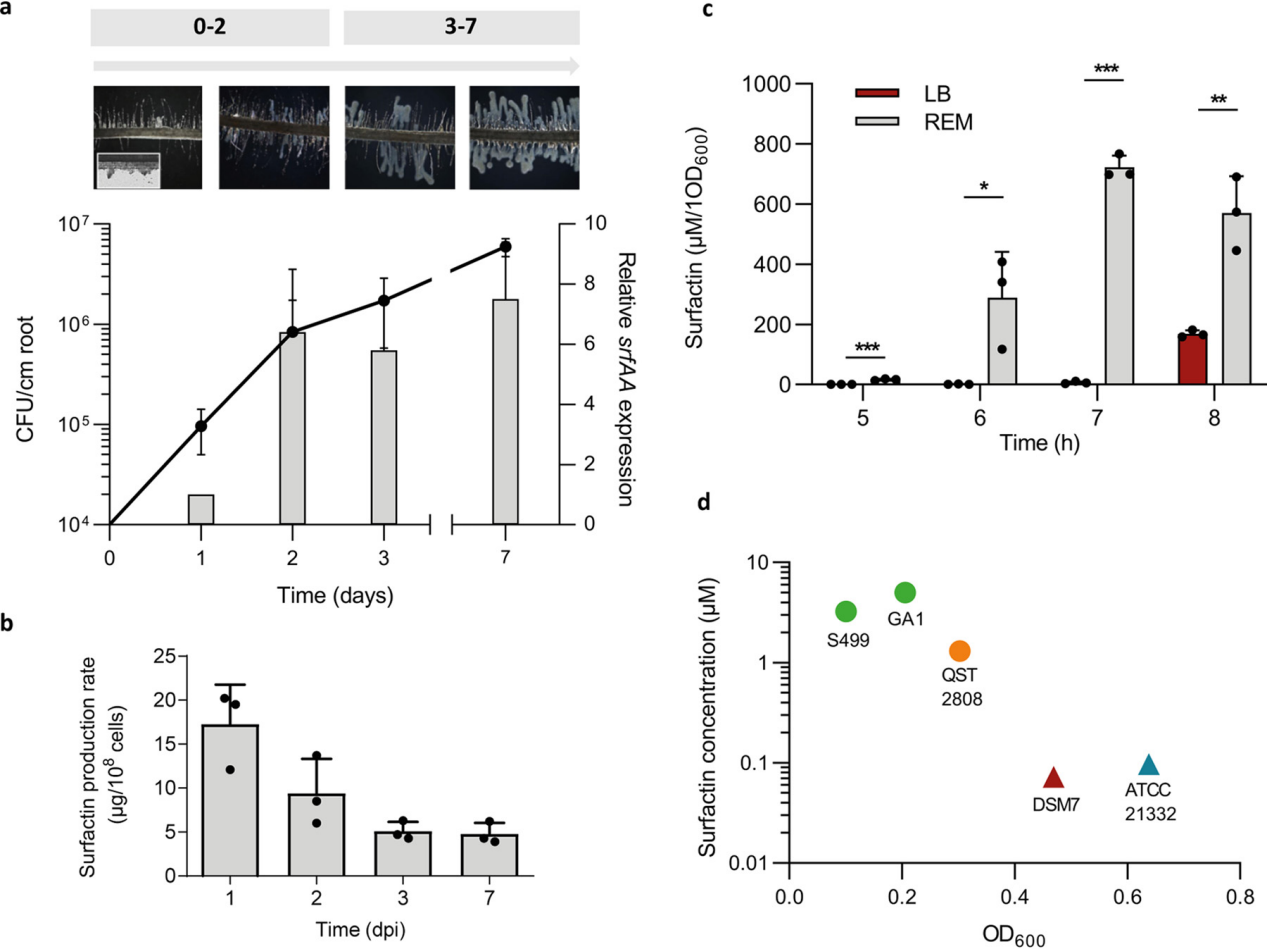
Impact of the specific rhizosphere nutritional context on early surfactin production. (a) Evaluation of bacterial population (black line, left axis) and relative *srfAA* expression on roots (gray bars, right axis) in a time frame of 7 days postinoculation (dpi). *Bacillus* progression on roots characterized by a biofilm formation was assessed by microscopy at each time point (top part). (b) Surfactin production rate on roots. Means ± SE from three biological replicates of one experiment are shown. (c) Surfactin accumulation measured by UPLC-MS in a 8-h time course experiment in REM medium (gray bars) compared with that in LB medium (red bars). Means ± SE from three biological replicates of one experiment are shown ***, *P* < 0.001; **, *P* < 0.01; *, *P* < 0.05. (d) Comparison of early surfactin accumulation (μM of surfactin on *y* axis linked to OD_600_ on *x* axis) in different *Bacillus* species, including *B. velezensis* (GA1 and S499 in green), *B. pumilus* (QST 2808 in orange), *B. amyloliquefaciens* (DSM 7 in red), and B. subtilis (ATCC 21332 in blue). Circle symbols represent plant-associated bacteria, whereas triangle symbols represent non-plant-associated bacteria.

Bacillus velezensis quickly colonizes tomato plantlets in a gnotobiotic system and forms visible biofilm-like structures covering the main root and embedding lateral roots after 24 to 48 h postinoculation (Fig. 2a). This process is correlated with consistent *srfAA* gene expression and surfactin production rate in the cell population at these early times, but it was maintained, albeit to a lower level, over the investigated time frame of 7 days (Fig. 2a and b). Since surfactin enhancement linked to the perception of the pectin backbone is only transient (Fig. 1d), we hypothesized that root exudates, constantly secreted by the plant, may also positively impact the synthesis of the lipopeptide. Surfactin production rate was thus compared upon growth in a classical laboratory medium (LB) and in a root exudate-mimicking medium (REM) reflecting the content of carbohydrates typically released by tomato or tobacco roots (27). It revealed an earlier and higher production by cells growing in REM (Fig. 2c). Surfactin production in REM is initiated earlier and is more efficient in *B. velezensis* than that in other closely related but non-plant-associated species, such as *B. amyloliquefaciens* or B. subtilis (Fig. 2d).

**FIG 3:**
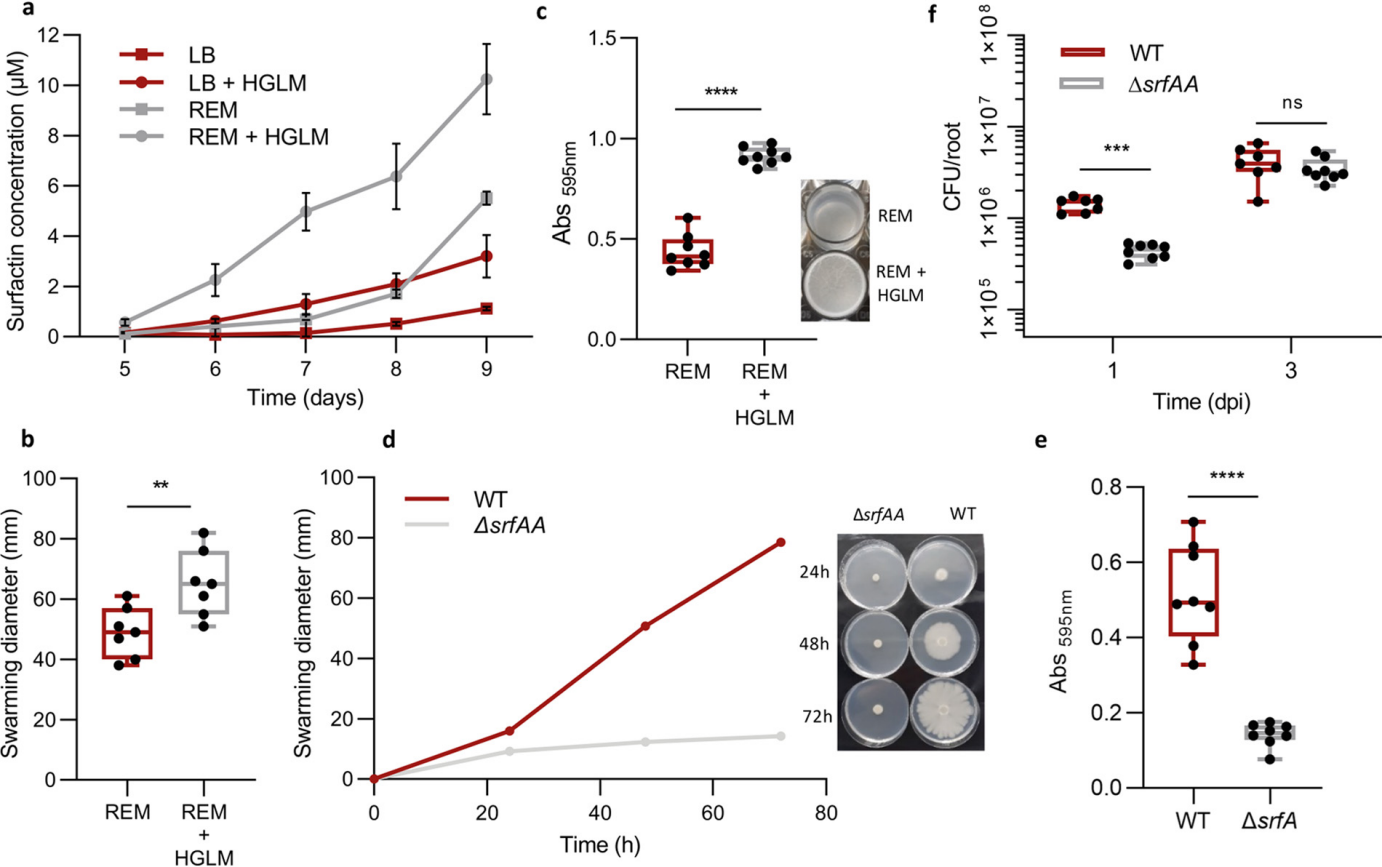
Ecological importance of an early surfactin accumulation. (a) Evaluation of HGLM and root exudate synergistic effect on early surfactin production. Time course experiment for surfactin quantification was performed in REM (gray curves) and LB medium (red curves) with (circle symbols) or without (square symbols) addition of HGLM. Means ± SE from three biological replicates of one experiment are shown. (b) Swarming potential of *B. velezensis* GA1 on soft agar plates after addition of HGLM or not. The box plots encompass the 1st and 3rd quartile, the whiskers extend to the minimum and maximum points, and the midline indicates the median (*n* = 7 biological replicates of one experiment). (c) Evaluation of *B. velezensis* ability to form pellicles on microwell plates after addition of HGLM or not. The box plots encompass the 1st and 3rd quartile, the whiskers extend to the minimum and maximum points, and the midline indicates the median (*n* = 8 biological replicates of one experiment). Pellicle formation is illustrated on the right. (d) Comparison of *B. velezensis* GA1 WT (red) and a Δ*srfAA* mutant (gray) for their swarming potential in a time course study. Means ± SE from three biological replicates of one experiment are shown. Time course study is illustrated on the right. (e) Comparison of pellicle formation between GA1 WT strain (red) and a Δ*srfAA* mutant (gray). The box plots encompass the 1st and 3rd quartiles, the whiskers extend to the minimum and maximum points, and the midline indicates the median (*n* = 8 biological replicates of one experiment) ****, *P* < 0.0001. (f) *In vitro* comparison of root colonization ability of GA1 (red boxes) and GA1 Δ*srfAA* (gray boxes) on tomato plantlets. The box plots encompass the 1st and 3rd quartiles, the whiskers extend to the minimum and maximum points, and the midline indicates the median (*n* = 7 biological replicates of one experiment) ***, *P* < 0.001; ns, nonsignificant.

Addition of HGLM in REM compared with LB revealed a cumulative effect of this PCWP and root exudates on surfactin production (Fig. 3a). This effect could be of clear ecological benefit for the bacterium since surfactin is known to favor the motility of multicellular communities and biofilm formation (16, 28, 29). However, a recent study questioned the real role of surfactin in these key functions since its production appears as nonessential for pellicle biofilm formation in B. subtilis NCIB 3610, suggesting a strain-dependent role (30). We reported previously that motility and biofilm formation are boosted upon growth on root exudates (27). Here, we show that HGLM supplementation also favors *B. velezensis* GA1 spreading on low-agar medium (Fig. 3b) and early biofilm formation based on pellicle development at the air-liquid interface (31) (Fig. 3c). The role of surfactin in swarming, pellicle formation, and early root colonization was further confirmed for *B. velezensis* GA1. Indeed, swarming motility on low-agar plates was almost reduced to zero in a surfactin-deficient mutant, and the same mutant was more than 3 times less efficient at producing pellicles at the air liquid interface and at promptly colonizing tomato roots after 1 day postinoculation compared with the wild type (WT) (Fig. 3d, e, and f). Collectively, these data allow a correlation of the positive impact of PCWPs on bacterial motility, biofilm formation, and early root colonization through an anticipated surfactin production in *B. velezensis*.

### Surfactin induction by PCWPs is not linked to major transcriptional changes.

**FIG 4:**
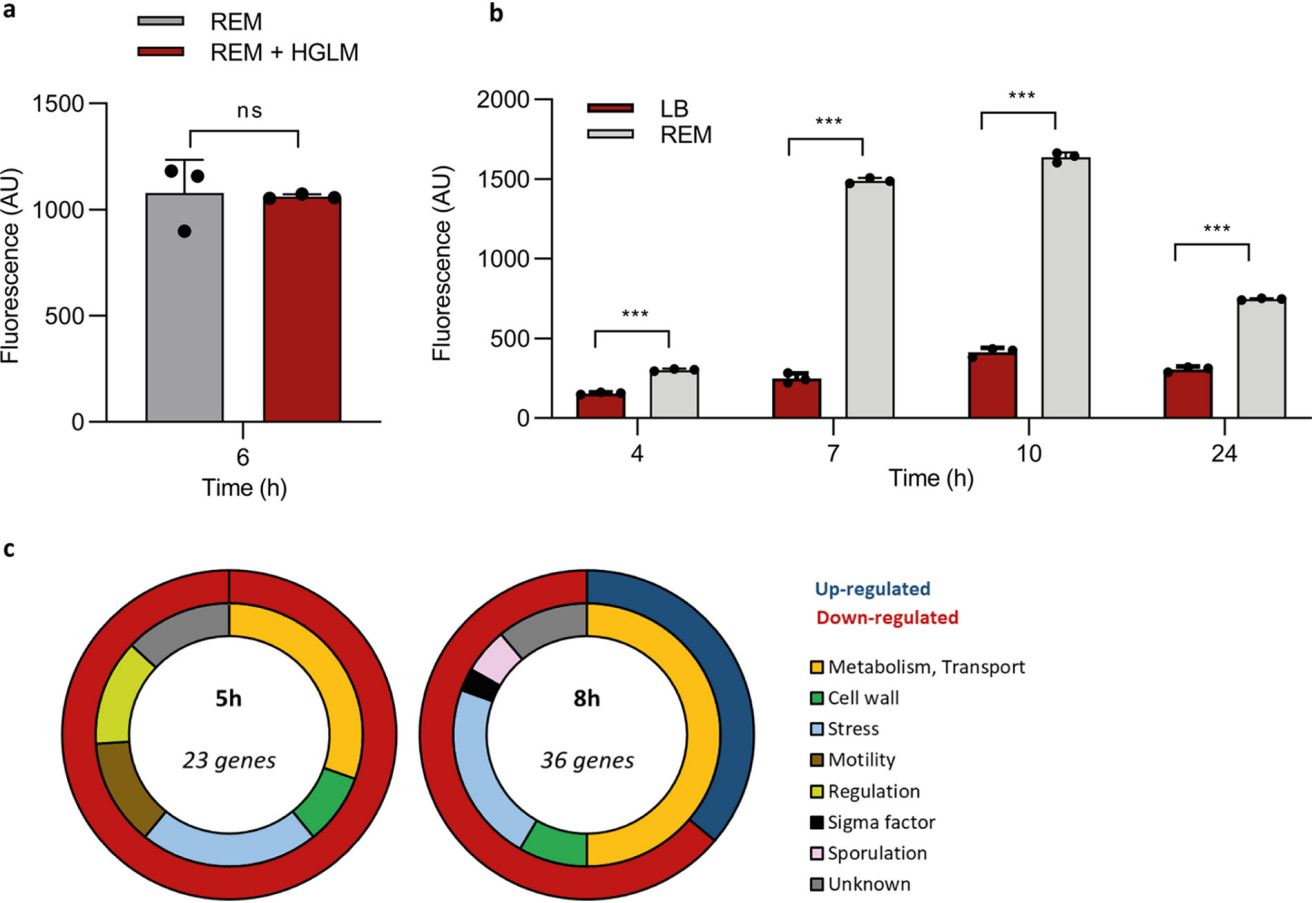
Impact of plant trigger perception on *Bacillus* transcriptome. (a) Surfactin expression measured by fluorescence in the GA1 P*srfA_gfp* reporter strain at early-exponential phase in REM (gray bars) compared with that of REM supplemented with HGLM (red bars). Means ± SE from three biological replicates of one representative experiment are shown; ns, nonsignificant. (b) Surfactin expression measured by fluorescence in the GA1 P*srfAp::gfp* reporter strain in a 24-h time course study in EM (gray bars) compared with that in LB medium (red bars). Means ± SE from three biological replicates of one representative experiment are shown. ***, *P* < 0.001. (c) Classification of the different genes carrying a significant fold change (1.5 log_2_) 5 and 8 h after addition of HGLM compared with that of the control condition. The outer circle represents the proportion of upregulated (dark blue) and downregulated (red) genes. The inner circle represents the proportion of genes belonging to the different functional family described in the legend.

Both HGLM and root exudates stimulate surfactin production in GA1. However, while no activation of the *srfA* biosynthetic gene cluster was observed upon HGLM addition (Fig. 4a), an early and high surfactin gene expression was measured in P*srfA_gfp* cells growing in REM compared with LB medium (Fig. 4b), suggesting that the two phenomena rely on a different regulatory pathway. To unravel transcriptome-wide changes in GA1 associated with the perception of HGLM, RNA sequencing was performed on cells grown in REM with or without addition of HGLM and collected at various time points (lag, early-exponential, and a mid-exponential phases). The data confirmed that HGLM perception is not linked to an increased expression of the *srfA* operon but also revealed a quite limited and transient transcriptional reprogramming with only 58 genes differentially expressed over this time frame (Table 1). Remarkably, more than 30% of these genes are involved in stress response or cell wall modifications and are downregulated in the presence of HGLM (Fig. 4c). We thus hypothesize that a long-term coevolution process may have facilitated *Bacillus* establishment on the roots by the inhibition of a costly stress response after perception of HGLM. Addition of HGLM also leads to a 4.2-fold reduced expression of *flgM* encoding an inhibitor of SigD, the σ factor involved in the activation of motility-related genes (32). This process may contribute to an enhanced spreading of multicellular communities in addition to the positive effect of surfactin mentioned above.

**TABLE 1:** Differentially expressed genes in *B. velezensis* GA1 after HGLM perception

Locus tag	Name			Conservation in related species^*a*^				Category	Information
		Fold change at:		*B. amyloliquefaciens* DSM 7		B. subtilis 168			
		5 h	8 h	QC (%)	ID (%)	QC (%)	ID (%)		
GL331_00010	*floT* (*yuaG*)		−3.048	100	93.2	97	81.46	Stress	Inner membrane protein, flotillin-like protein
GL331_00015	*yuaF*	−3.016		99	93.4	94	68.97	Stress	Involved in the control of membrane fluidity
GL331_00985	*yusU*	−3.072		100	95.12	99	76.37	Unknown	Unknown
GL331_01085	*liaH*		−4.878	100	93.95	100	77.21	Stress	*lia* operon expression modulator, similar to phage shock protein, resistance against oxidative stress, and cell wall antibiotics
GL331_01090	*liaI*		−3.846	100	93.96	100	69.71		Resistance against oxidative stress and cell wall antibiotics
GL331_01180	*fhuD*	−3.176		100	95.1	99	77.31	Transport	Hydroxamate siderophore ABC transporter
GL331_01520	*opuCA*		3.066	100	95.61	99	82.35	Transport	Glycine betaine/carnitine/choline/arsenobetaine/arsenocholine ABC transporter
GL331_02210	*hpf*	−4.636		100	97	100	83.71	Stress	General stress protein, required for ribosome dimerization in the stationary phase, protects essential ribosomal proteins
GL331_02265	*flgN*	−3.608		100	96.48	76	76.55	Motility	Flagellar filament assembly protein
GL331_02270	*flgM*	−4.334		100	98.5	100	77.15		Negative regulator of flagellin synthesis, anti-sigD
GL331_02275	*yvyF*	−4.562		100	95.71	100	78.38		Unknown
GL331_02335	*tuaF*		−5.05	100	92.51	100	70.91	Cell wall	Teichuronic acid biosynthesis protein
GL331_02355	*tuaB*		−3.262	100	94.41	100	73.25	Cell wall	Polymer export
GL331_03100	*maeA*		−3.69	100	97.29	100	82.88	Metabolism	Malate utilization
GL331_03645	*ywcE*		−3.516	100	96.93	89	85.04	Sporulation	Holin, spore morphogenesis and germination protein
GL331_03950	*cydB*	−5.742	−3.506	100	95.38	100	79.45	Metabolism	Cytochrome d ubiquinol oxidase subunit 2
GL331_03955	*cydA*	−4.792		100	96.52	100	82.3		Cytochrome d ubiquinol oxidase subunit 1
GL331_04110			−3.374					Unknown	Hypothetical protein
GL331_04120	*wapA*		−4.134			92	77.81	Cell wall	Cell wall-associated protein precursor
GL331_04135	*yxiE*	−3.784		100	95.53	98	78.25	Stress	Universal stress protein
GL331_04235		−3.672		100	94.18	89	75.79	Transport	Hydroxamate siderophore ABC transporter
GL331_04550			3.198	99	96.39			Transport	Transport protein (putative quinolone resistance)
GL331_04560	*thiF*		3.138					Metabolism	Thiazole biosynthesis adenylyltransferase
GL331_04675			−3.768	100	92.69			Unknown	PIG-L family deacetylase
GL331_05550	*ctsR*	−3.034		100	96.77	100	86.45	Regulation	Transcriptional repressor, protein synthesis, modification and degradation
GL331_06040	*btr*	−3.352		100	91.23	72	68.27	Regulation	Transcriptional activator, regulation of iron acquisition
GL331_06530	*yceF*		−3.108	100	96.77	100	82.43	Stress	General stress protein, manganese resistance protein
GL331_06540	*yceH*		−3.104	100	95.22	98	83.68		Similar to toxic anion resistance protein
GL331_06585		−3.342		100	94.65	97	81.42	Metabolism	l-Lactate dehydrogenase
GL331_07095	*yczJ*		−4.126	100	95.64	99	74.38	Unknown	Unknown
GL331_07100			−3.124						
GL331_07310	*gsiB*	−3.068		100	96.6	93	89.97	Stress	General stress protein, response to glucose starvation or to water deficits
GL331_08210	*pspA*		−3.664	100	93.74	96	74.52	Stress	Phage shock protein A homolog, paralogous to *liaH*
GL331_08215	*ydjG*		−3.246	99	94.43	99	75.05		Unknown
GL331_08225	*ydjI*		−3.014	99	97.01	98	77.93		Unknown
GL331_09875	*nhaX*	−3.04		99	92.59	99	74.9	Stress	Universal stress protein
GL331_10225	*yhzC*	−3.306		100	94.87	100	85.04	Unknown	Unknown
GL331_10530	*argJ*		3.852	99	94.82	99	77.23	Metabolism	Biosynthesis of arginine
GL331_10535	*argB*		5.39	100	91.51	100	71.5		
GL331_10540	*argD*		4.64	100	93.96	96	74.62		
GL331_10545	*carA*		5.726	100	93.48	100	73.46		
GL331_10715	*cwlQ*	−3.67		100	92.43	75	77.02	Cell wall	Bifunctional cell wall hydrolase
GL331_14205	*iseA*	−5.102		100	93.19	100	72	Cell wall	Cell wall endopeptidases and cell separation inhibitor
GL331_15495	*sigX*		−3.024	100	97.44	99	87.18	Sigma factor	RNA polymerase sigma factor
GL331_15575	*ribH*		3.352	100	95.91	100	81.29	Metabolism	Riboflavin biosynthesis
GL331_15580	*ribA*		3.946	100	94.57	100	78.11		
GL331_15585	*ribE*		3.896	100	94.29	100	74.88		
GL331_15590	*ribD*		4.102	100	94.44	99	76.73		
GL331_15930	*loaP*	−3.848						Regulation	Antiterminator involved in regulation of polyketide synthesis
GL331_16515	*pstBB*		−5.384	99	92.7	93	73.36	Metabolism, transport	High-affinity phosphate uptake, phosphate ABC transporter
GL331_16520	*pstBA*		−4.924	100	92.32	89	77		
GL331_16525	*pstA*		−4.038	100	94.8	99	79.07		
GL331_17335			−5.61	100	89.17	48	75.86	Unknown	Unknown
GL331_17345	*safA*		−4.26	99	89.73	42	76.04	Sporulation	Major organizer of the inner spore coat
GL331_17895	*pftB*	−3.294		100	95.03	100	81.73	Metabolism, transport	Pyruvate transporter
GL331_17900	*pftA*	−3.958		100	97.28	83	79.89		
GL331_18085	*ytzJ*	−3.29		100	98.96	100	84.38	Unknown	Unknown
GL331_18115	*argH*		5.002	100	95.06	99	83.98	Metabolism	Biosynthesis of arginine
GL331_18120	*argG*		5.144	100	95.3	100	81.77		

a QC, query cover; ID, identity.

### Root exudates drive the bacterium to form surfactin homologues with long fatty acid chain (LFAC) and variants enriched in valine.

**FIG 5:**
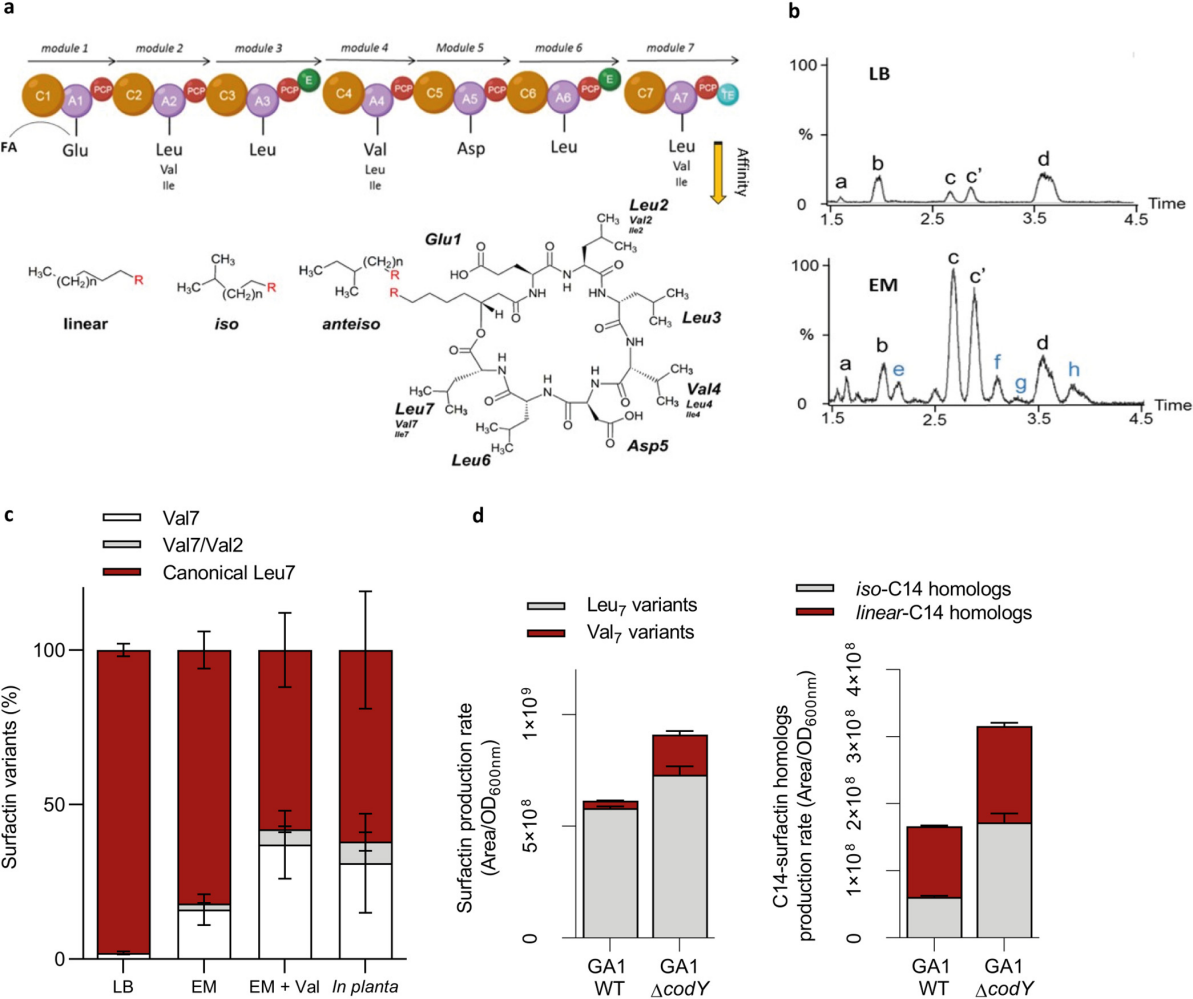
Qualitative impact of root exudates on surfactin production. (a) Representation of the NRPS machinery leading to the assembly of the surfactin molecule. This megaenzyme is organized in 7 functional units called modules which are each responsible for the incorporation of one amino acid building block into the growing peptide chain. Each module is subdivided into different domains, including an adenylation (A; violet circle) and a peptidyl carrier protein (PCP; red circle) catalyzing the peptide initiation and one condensation domain (C; brown circle) responsible for peptide elongation. The termination of the peptide synthesis is performed by a thioesterase domain (TE; blue circle) in the last module. Modules 3 and 6 also possess an epimerization domain (E; green circle). The surfactin molecule contains a 7-amino acid chain structured as follows: l-Glu–l-Leu–d-Leu–l-Val–l-Asp–d-Leu–l-Leu. In some specific variants, Leu in position 2 and/or 7 can be substituted by a Val and more rarely by an Ile, and inversely, Val in position 4 can be substituted by a Leu and also more rarely by a Ile. In addition to the amino acid chain variability, multiple homologues with the same peptidic core but differences in terms of fatty acid chain length (C_12_ to C_17_) or isomerization (iso, anteiso, or linear configuration) can also be produced. (b) Comparison of surfactin pattern in REM and LB medium. Based on MS-MS analyses, nine different surfactin forms were identified (a, C_12_-Glu-Leu-Leu-Val-Asp-Leu-Leu; b, C_13_-Glu-Leu-Leu-Val-Asp-Leu-Leu; c, iso-C_14_-Glu-Leu-Leu-Val-Asp-Leu-Leu; c’, *n-*C_14_-Glu-Leu-Leu-Val-Asp-Leu-Leu; d, C_15_-Glu-Leu-Leu-Val-Asp-Leu-Leu; e, C_13_-Glu-Leu-Leu-Val-Asp-Leu-Val; f, C_14_-Glu-Leu-Leu-Val-Asp-Leu-Val; g, C_14_-Glu-Leu-Leu-Val-Asp-Leu-Val; and h, C_14_-Glu-Val-Leu-Val-Asp-Leu-Val). (c) Relative proportions of surfactin variants in LB, REM, REM supplemented with valine, and *in planta.* (d) Qualitative and quantitative role of CodY on surfactin production. In a WT strain, 95% of the surfactin molecules are carrying a Leu in position 7 (gray bars) and only 5% are carrying a Val (red bars), whereas in the Δ*codY* mutant, almost 25% of the surfactin molecules are carrying a Val in position 7 and 75% are carrying a Leu. In addition, the amount of total surfactin production rate of 150% can be observed in the Δ*codY* mutant compared with the WT strain. Proportion of iso-C_14_ is also affected by CodY, 36% of total C_14_ are iso-fatty acid (gray bars), and 64% are linear (red bars) in WT strain, whereas in the Δ*codY* mutant, 55% of C_14_ are iso-C_14_ and 45% are linear. Again, the total amount of C_14_ is higher in the Δ*codY* mutant (increase of 190%).

The nonribosomal peptide synthetase assembly (NRPS) machinery works as an assembly line in which each module is responsible for recruiting and binding a specific amino acid to the nascent peptide after a first lipoinitiation step for binding the fatty acid (FA) taken up from the cellular pool (Fig. 1a) (33, 34). In that way, surfactin is typically composed of saturated C_12_ to C_19_-FA of the linear, iso, or anteiso type of branching (35). Besides an increased production of surfactin, we also observed an effect on the pattern of surfactin variants synthesized by *B. velezensis* in the presence of artificial plant exudates, as well as in naturally produced exudates and *in planta* upon root colonization (see Fig. S4 in the supplemental material). Indeed, ultraperformance liquid chromatography-mass spectrometry (UPLC-MS) profiling revealed that the surfactin pattern produced by GA1 in REM is enriched in surfactin iso-C_14_ (*i*C_14_) and other variants compared with that in LB medium (Fig. 5b). They correspond to variants of the canonical structure with substitution of Leu by Val for the last residue of the cyclic peptide moiety (Val_7_) and, to a much lower extent, to the same substitution in position 2 (Val_2_) (Fig. 5c; see Fig. S5 in the supplemental material). Valine is used both as a precursor for the synthesis of branched fatty acids with an even number of carbons and as a building block by the NRPS to form the peptide moiety. Supplementation of the medium with deuterated l-Val-d^8^ resulted in an additional increase in the proportions of surfactin iso-C14 and Val_7_ isoforms labeled at the expected positions in the peptide and in the fatty acid tail (Fig. S5). Based on these data, the higher relative proportions of *i*C_14_Val^7^ formed in REM, but also *in planta* (Fig. 5c), most probably result from some enrichment of the intracellular pool in valine upon growth in the presence of root exudates (see Discussion in the supplemental material; see Fig. S6 in the supplemental material). Given the reduced specificity of NRPS domains involved in selection and activation of leucine at positions 2 and 7, the megaenzyme would preferably bind valine as it is more available in the pool.

10.1128/mBio.01774-21.4 FIG S4 UPLC-MS 3D representation of *B. velezensis* surfactin pattern diversity produced in REM (a), in natural root exudates (b), or *in planta* (c). The *x* axis indicates the retention time (min), the *y* axis the mass/charge ratio (*m/z*), and the *z* axis the peak intensity (AU). Each blue peak represents a surfactin homologue. Download FIG S4, TIF file, 0.6 MB. Copyright © 2021 Hoff et al. 2021 Hoff et al. https://creativecommons.org/licenses/by/4.0/ This content is distributed under the terms of the Creative Commons Attribution 4.0 International license.

10.1128/mBio.01774-21.5 FIG S5 Surfactome variability. (a and b) High-resolution tandem mass spectrometry (HR-MS/MS) analyses. (a) Schematic representation of surfactin fragmentation. 1, surfactin Leu^7^; 2, surfactin Val^7^. (b) List and mass error of detected y-ions after fragmentation of surfactins produced in EM for C_13_ to C_15_ Leu^7^ and Val^7^ surfactins. Impact of medium supplementation with deuterated l-Val-d^8^ on *B. velezensis* surfactome. Precursor feeding with 8 time deuterated valine will result in a mass increment of 7 mass unit in iso-even fatty acid (i.e., iso-C_14_; see insert) due the loss of α-deuterium during the transamination step. Fragmentation of iso-C_14_ surfactin shows a high proportion of deuterated b1 and b1-H_2_O fragment (*m/z*, 363 and 345, respectively). A small proportion of nondeuterated b1 and b1-H_2_O is also visible in the spectrum (*m/z*, 356 and m/z = 338, respectively). Download FIG S5, TIF file, 0.3 MB. Copyright © 2021 Hoff et al. 2021 Hoff et al. https://creativecommons.org/licenses/by/4.0/ This content is distributed under the terms of the Creative Commons Attribution 4.0 International license.

10.1128/mBio.01774-21.6 FIG S6 Nonribosomal biosynthesis via NRP synthetases and regulation by pleiotropic transcription factors, such as CodY, both drive the diversity and ratio of the surfactin precursors in the intracellular pool (i.e., branched-chain amino acids and fatty acids, blue panel). In turn, relative amounts in the intracellular pool (green panel) as well as the selectivity of each adenylation domain regarding the type of amino acid it activates (orange panel) lead to the production by the NRP machinery of different surfactin variants (gray panel) detected in reversed phase LC-MS (yellow panel). Download FIG S6, TIF file, 0.3 MB. Copyright © 2021 Hoff et al. 2021 Hoff et al. https://creativecommons.org/licenses/by/4.0/ This content is distributed under the terms of the Creative Commons Attribution 4.0 International license.

As already described in B. subtilis (36, 37), the pleiotropic regulator CodY acts as repressor of surfactin synthesis in *B. velezensis* GA1 as illustrated by the 1.9-fold increase in production by the Δ*codY* mutant of strain GA1. Interestingly, CodY activity/*codY* expression is also itself impacted negatively by high cellular concentrations in branched-chain amino acids (38). Both quantitative and qualitative changes in surfactin production upon growth in exudates could therefore be, at least partly, due to a lower CodY activity (see Text S2 in the supplemental material). In support of the role played by this regulator, a similar impact on surfactin pattern was observed by deleting *codY* in GA1 or by supplementing the culture medium of the wild-type with valine (Fig. 5d).

10.1128/mBio.01774-21.9 TEXT S1 Quantitative RT-PCR, size exclusion chromatography (SEC-MALLS), pectate lyase activity measurement, and surfactin structural elucidation. Download Text S1, DOCX file, 0.02 MB. Copyright © 2021 Hoff et al. 2021 Hoff et al. https://creativecommons.org/licenses/by/4.0/ This content is distributed under the terms of the Creative Commons Attribution 4.0 International license.

10.1128/mBio.01774-21.10 TEXT S2 Supplementary discussion: surfactin diversity. Download Text S2, DOCX file, 0.03 MB. Copyright © 2021 Hoff et al. 2021 Hoff et al. https://creativecommons.org/licenses/by/4.0/ This content is distributed under the terms of the Creative Commons Attribution 4.0 International license.

### Long fatty acid chain surfactins act as key triggers of receptor-independent plant immunity.

Based on the potential of surfactin to serve as a host immunity elicitor (9, 39), we next wanted to evaluate the possible relevance of quantitative and qualitative modulation of the surfactin pattern driven by the plant for its own benefit.

**FIG 6:**
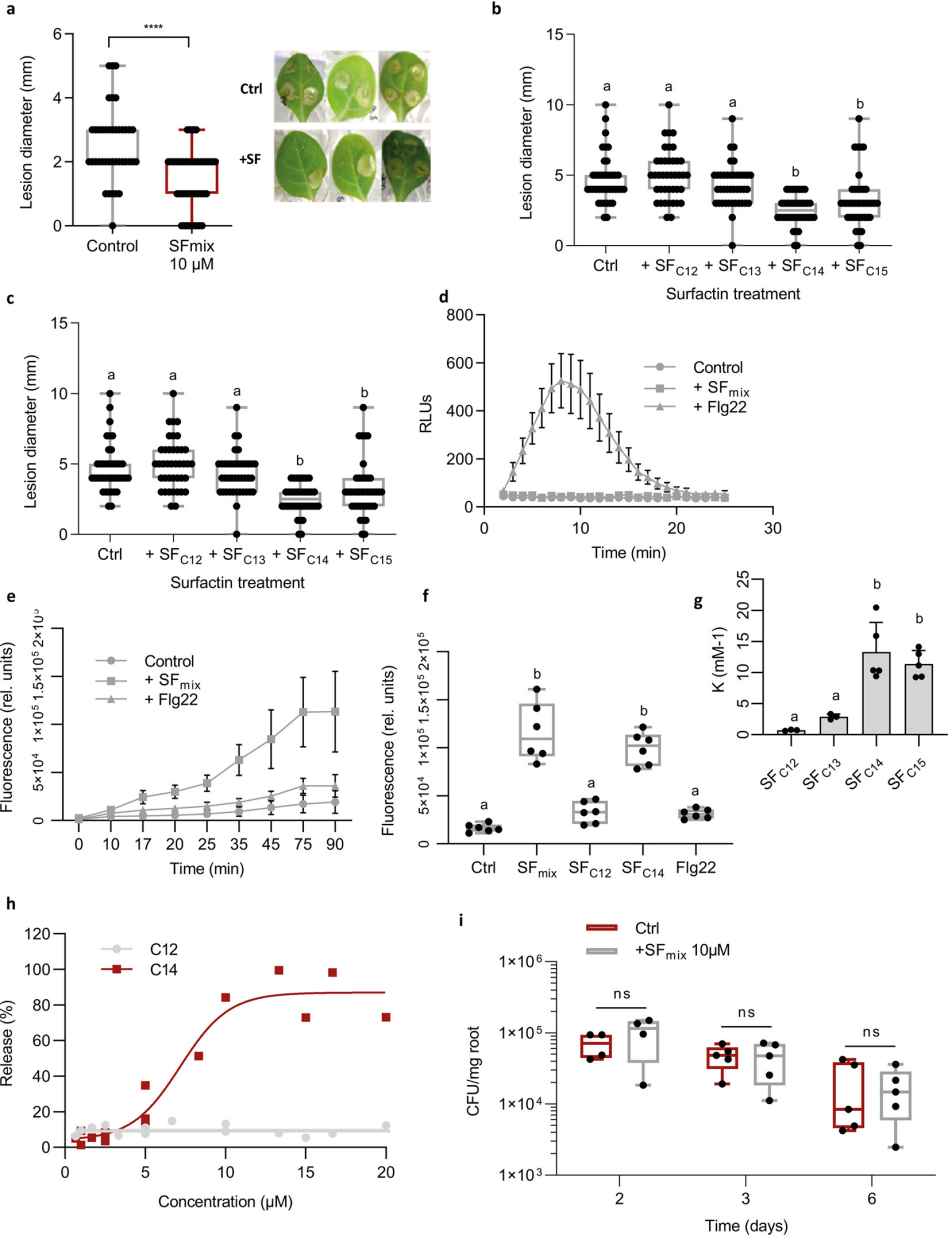
Impact of surfactin homologues on *Solanaceae* plant immunity. (a to c) Systemic resistance induced in hydroponically grown tobacco by surfactin and expressed as reduction of *B. cinerea* infection (illustration of the reduction in the diameter of spreading lesions on infected leaves) in plants treated at the root level prior to pathogen inoculation on leaves compared with that of control plants. Data represent results grouped from 2 independent experiments with similar results and each involving 5 plants with 4 lesions on the second leaf (*n* = 40). The box plots encompass the 1st and 3rd quartiles, the whiskers extend to the minimum and maximum points, and the midline indicates the median (*n* = 7 biological replicates of one experiment). (a) Effect of surfactin homologues (SF mix) as naturally coproduced by the bacterium (C_12_/C_13_/C_14_/C_15_ in relative proportions of 8%/17%/33%/42%); ****, *P* <0.0001. (b) Effect of HPLC-purified surfactin homologues applied at 10 μM with fatty acid chains from C_12_ to C_15_. Significant difference between each condition is indicated by different letters; *P* < 0.05. (c) Effect of the most active C_14_ homologue tested at various concentrations. The significant difference between each condition is indicated by different letters; *P* < 0.05. (d and e) Stimulation of oxidative burst in root tissues upon treatment with an SF mix and to the response observed by treating roots with flagellin (flg22; 1 μM) used as a positive control. (d) Stimulation of apoplastic ROS accumulation (DCFH-DA fluorescent probe) in root tissues upon treatment with a surfactin mix applied at 15 μM. Means and standard deviations are shown for one representative experiment performed on nine samples per treatment, with each containing three root segments (approximately 100 mg FW) collected from different plants (*n* = 9). A similar trend was obtained in an independent assay. (e) Stimulation of cytoplasmic hydrogen peroxide production in root cells. Means and SDs were calculated from measurements performed on three samples per treatment, with each containing three root segments (approximately 100 mg FW) collected from different plants. Data represent values obtained from two independent experiments (*n* = 6 per treatment). (f) Stimulation of cytoplasmic hydrogen peroxide production in root cells after treatment with C_12_ and C_14_ surfactin homologues as a representative of short and long fatty acid chains, respectively. Flg22 was used as a control. The box plots encompass the 1st and 3rd quartiles, the whiskers extend to the minimum and maximum points, and the midline indicates the median (*n* = 6 biological replicates of one experiment). The significant difference between each condition is indicated by different letters; *P* < 0.0001. (g) Binding coefficient (K) of surfactin homologues (C_12_ to C_15_) to large unilamellar vesicles (LUV) composed by PLPC-sitosterol-glucosylceramide (60:20:20 molar ratio). Means ± SE from three to five biological replicates of one representative experiment are shown. The significant difference between each condition is indicated by different letters; *P* < 0.05. (h) Release of 8-hydroxypyrene-1,3,6 trisulfonic acid (HPTS) from PLPC-sitosterol-glucosylceramide (60:20:20 molar ratio) LUV, upon addition of surfactin C_12_ or C_14_ at different concentrations. The ordinate shows the amount of HPTS released after 15 min in the presence of the C_12_ or C_14_ as a percentage of the total amount released by Triton X-100. (i) Influence of roots pretreatment with 10 μM surfactin (blue boxes) compared with that of nontreated roots (red boxes) on *B. velezensis* GA1 root colonization. The box plots encompass the 1st and 3rd quartiles, the whiskers extend to the minimum and maximum points, and the midline indicates the median (*n* = 5 biological replicates of one experiment). Ns, nonsignificant.

Upon application as a root treatment, pure surfactin used as a mixture of isoforms formed in REM induced systemic resistance in hydroponically grown tobacco plants providing approximately 45% to 50% significant disease reduction on leaves infected subsequently with the pathogen *Botrytis cinerea* (Fig. 6a). The various isoforms were then HPLC purified and tested individually revealing that only long fatty acid homologues (C_14_/C_15_) provided systemic protection to a similar level, whereas short fatty acid homologues (C_12_/C_13_) were inactive (Fig. 6b). Moreover, plant immunization by surfactin is dose dependent, and concentrations up to 5 μM are sufficient to significantly stimulate ISR (Fig. 6c). Interestingly, such low μM concentrations are actually in the range of those that could accumulate in the root vicinity within a few days upon colonization by GA1 (see Fig. S7 in the supplemental material).

10.1128/mBio.01774-21.7 FIG S7 Estimated surfactin concentration surrounding the rhizoplan in a solid matrix. Surfactin was quantified based on the amounts measured by UPLC-MS in three extracts (mean ± SD); each were prepared from 3 roots and surrounded by gelified medium from 3 individual plantlets. Surfactin concentration (μM) was calculated based on the mean value. Download FIG S7, TIF file, 0.03 MB. Copyright © 2021 Hoff et al. 2021 Hoff et al. https://creativecommons.org/licenses/by/4.0/ This content is distributed under the terms of the Creative Commons Attribution 4.0 International license.

We next wanted to correlate this systemic protection induced by the lipopeptide with its potential to trigger locally early immune-related events, such as the extracellular burst, in reactive oxygen species (ROS) involved in defense and signaling in pathogen-triggered immunity (PTI) (40, 41). In contrast with flagellin (epitope Flg22), one of the best characterized microbe-associated molecular patterns (MAMPs) isolated from bacterial pathogens, treatment with surfactin did not induce burst in apoplastic ROS in root tissues (Fig. 6d). However, surfactin-mediated ROS signaling still occurs since a clear cytoplasmic ROS accumulation was observed (Fig. 6e). Little information is available about the spatiotemporal dynamics of such a ROS burst, but it may originate from different organelles and has been occasionally described in response to the perception of biotic and abiotic stresses (42, 43). Using cytoplasmic ROS as markers, the same trend as for ISR tests could be observed regarding the influence of the structure on the activity of surfactin since long fatty acid homologues but not short ones efficiently stimulated early immune reaction (Fig. 6f). This result means that a single additional methylene group in the fatty acid tail of the molecule (C_14_ versus C_13_) likely determines its immunization potential (Fig. 6b and f). In contrast, substitution of Leu^7^ by a Val in the C_14_ homologue does not impact activity, suggesting that the peptide moiety is not essential for perception by plant cells. In addition, the μM concentrations required for optimal eliciting activity of surfactin are very high compared with PAMPs active in the nM range (44). Our previous data showed that surfactin elicitation is still active after pretreatment of plant cells with proteases, strongly suggesting that the lipopeptide is not bound in the apoplast by some domain of a protein anchored in the plasma membrane. Moreover, the effect of surfactin is conserved when tested a few minutes after a first application of the molecule. By contrast with MAMPs, there is thus no refractory state due to saturation of high-affinity binding sites on putative receptors (45, 46). Collectively all these results indicate that surfactin is perceived by plant cells via a mechanism independent of pattern-recognition receptors (PRRs) involved in MAMP perception (40, 41, 44, 47, 48). We therefore postulated that surfactin perception relies on some interaction with the lipid phase of the plant plasma membrane. Binding experiments via isothermal titration calorimetry and leakage assays based on the release of fluorescent probe were performed using liposomes prepared with lipids specific to the plant plasma membrane (palmitoyl linoleoyl phosphatidylcholine [PLPC]/sitosterol/glucosylceramide). It revealed that long fatty acid homologues have a higher affinity for these vesicles than the short fatty acid forms and display a higher destabilizing effect on the lipid bilayer when added at concentrations of 5 μM or higher (Fig. 6g and h). These biophysical data thus correlated well with the contrasting biological activities of longer C_14_/C_15_ and shorter C_12_/C_13_ surfactin homologues.

According to the priming concept (49), we previously showed that ISR triggered by the lipopeptide in that plant as well as in tobacco and *Arabidopsis* is not associated with a fast and strong expression of defensive mechanisms before pathogen infection (20, 39). In order to verify that surfactin elicitation does not cause a massive release of antimicrobials from plant tissues, tomato roots were pretreated with the lipopeptide before inoculation with *B. velezensis*. As expected, it did not impact the subsequent colonization in terms of rate and dynamics compared with untreated plants, indicating the absence of potential adverse effects on the bacterial partner (Fig. 6i).

## DISCUSSION

A large part of the interactions between bacteria and plants is known to be mediated by small-size secreted products (50). However, a better understanding of the chemical cross talk at the plant-bacterium interface and its impact on bacterial ecology, plant fitness, and immune responses remains challenging. In epiphytic soil bacilli, root exudates induce expression of an array of genes involved in various functions, such as chemotaxis and nutrient acquisition (51–53). Our data further illustrate that the use of this cocktail of molecules released by roots and also the perception of some cell wall polymers may drive these bacteria to efficiently produce key components of the secondary metabolome and more specifically the multifunctional surfactin lipopeptide (20). As an amphiphilic molecule and powerful biosurfactant, surfactin is presumably viewed as a membrane-active compound with potent antimicrobial activity. However, this lipopeptide is poorly antibacterial and antifungal (54). In *B. velezensis*, more obvious ecological functions of this CLP are to contribute to motility, biofilm formation, and root colonization. An enhanced production upon host perception thus constitutes a major force driving successful rhizosphere establishment.

Homogalacturonan acts as a cue to enhance surfactin secretion by bacterial cells, but no transcriptional induction of the corresponding biosynthesis operon was observed. Surfactin synthesis is integrated in a complex network involving several pleiotropic regulators acting directly or indirectly on the expression of the *srfA* operon (55–58). However, we hypothesize that surfactin induction by HGLM may rather rely on posttranscriptional changes as reported for the effect of the DegU and YczE regulators on the production of another CLP, bacillomycin D (59). Despite the relatively close genetic proximity between the tested strains, our data showing a variable level of surfactin induction in response to HGLM suggest that regulation of surfactin may be slightly different in *B. velezensis*, *B. amyloliquefaciens*, and B. subtilis. As it represents a key infochemical devoted to cross talk with the host plant, surfactin regulation may have been fine-tuned in rhizosphere species to better fit with the nutritional or more broadly the ecological context.

Deciphering the mechanism by which *B. velezensis* recognizes pectin and enhances surfactin production would help to identify candidate genes and pathways that are responsible for plant sensing, ensuring persistence on roots which globally remains very poorly known for beneficial rhizobacteria. We are currently investigating whether some cell surface proteins may act as receptors for homogalacturonan perception and binding, as described recently for *Sphingomonas* sp. (60), another beneficial species living in association with plants (61). Some insights could be obtained by scrutinizing the few genes conserved in *B. velezensis* but missing in non-plant-associated *B. amyloliquefaciens* strains that are not responsive to pectin (62). Interestingly, shorter fragments of HG and galacturonic acid do not stimulate surfactin secretion. It is therefore tempting to hypothesize that sensing an unaltered polymer could indicate a healthy host suitable for bacterial colonization, while the perception of monomers or low DP oligomers may reflect a dead or infected plant that is unable to adequately provide resources.

Our data illustrate for the first time that *B. velezensis* can also modulate qualitatively its surfactin pattern by growing in its natural nutritional context, i.e., on root exudates. Substitution of leucine by valine in the peptide part is not expected to impact the contribution of the lipopeptide to colonization by the producing strain itself, considering the minor effect of these structural changes on motility and biofilm formation potential (18). Small modifications in the peptide sequence may nevertheless avoid surfactin hijacking for use as a signal prompting heterologous biofilm formation by closely related competitor species (18). Based on our observations, the most obvious benefit of an increased proportion of long fatty acid chain homologues is for the host plant since they represent the most active forms for priming immunity with no impact on host fitness (20, 39), in contrast with PTI (63, 64). As the bacterial partner does not have to face strong defensive responses from this reaction, it ensures positive mutualistic cohabitation allowing establishment of populations on roots. The persistence of threshold populations is necessary for the consistent production of other specialized secondary metabolites more directly involved in warding off both microbial competitors and plant soilborne pathogens in the context of biocontrol.

Surfactin stimulation upon sensing host molecular patterns may thus reflect an aspect of plant-*Bacillus* coevolution, as it makes a shared good out of this multifunctional lipopeptide. To some extent, it might represent a facet of the plant-driven selection process resulting in active recruitment of this bacterium as a species that provides beneficial functions. Other bacterial genera, such as Pseudomonas also prevailing in the rhizosphere microbiome, actively produce CLPs with similar roles as surfactin. Evaluating whether their synthesis is also modulated by plant cues would conceptually allow broadening the significance of these lipopeptide-mediated interkingdom interactions for bacterial ecology, plant health, and biocontrol.

## MATERIALS AND METHODS

### Bacterial media and growth conditions.

Cultures were performed at 26°C in root exudate mimicking medium (EM) (27) or in LB medium. EM was prepared by mixing 3 different solutions (pH 7.5) after autoclaving, as follows: 1/4 of sugar solution (per liter of 4 g glucose, 6.8 g fructose, 0.8 g maltose, and 1.2 g ribose), 1/2 of organic acid solution (per liter of 8 g citrate, 8 g oxalate, 6 g succinate, 2 g malate, and 2 g fumarate), and 1/2 of all media [per liter of 0.685 g KH_2_PO_4_, 21 g morpholinepropanesulfonic acid (MOPS), 0.5 g MgSO_4_ 7H_2_O, 0.5 g KCl, 1 g yeast extract, 1 g Casamino Acids, 2 g (NH_4_)_2_SO_4_, and 100 μl of each trace solution of Fe_2_ (SO_4_)_3_ (12 g/liter^−1^), Mn SO_4_ (4 g/liter^−1^), Cu SO_4_ (16 g/liter^−1^), and Na_2_ MoO_4_ (40 g/liter^−1^]). To test the effect of plant cell wall polymers, each specific plant polysaccharide was added at a final concentration of 0.1% in the culture medium. Low (HGLM, <5%) and high (HGHM, >95%) methylated homogalacturonan were provided from Elicityl Oligotech, whereas oligogalacturonides and d-galacturonic acid were provided from Sigma.

### Strain construction.

All the bacterial strains used in this study are listed in Table 2. All the primers used in this study are available upon request. To follow the expression level of the *srf* operon in GA1, we constructed a *gfp* transcriptional fusion under the control of the *srf* promoter and integrated it into the *amyE* locus. First, a GA1 *amyE* amplicon containing a native KasI restriction site was integrated in the PGEMT easy system. In parallel, a *cat*-*gfp* cassette containing (i) a chloramphenicol resistance gene (*cat*) and (ii) a promoterless *gfpmut3.1* gene was amplified with primers containing KasI sites at their 5′ extremities using the pGFP star as a matrix (65). The pGEMT *amyE* plasmid and the *cat*-*gfp* amplicon were both digested by KasI (New England BioLabs [NEB]), and the two linear fragments with compatible 5′ overhangs were ligated together to obtain the PGEMT *amyEup-cat*-*gfp-amyEdw* plasmid. To construct the final mutation cassette, an overlap extension PCR was assessed by following the method developed by Bryksin and Matsumura (66). One first fragment containing the upper *amyE* homologous region and the *cat* gene and a second fragment englobing the *gfpmut3.1* gene and the lower *amyE* homologous region were both amplified using the PGEMT *amyEup-cat*-*gfp-amyEdw* plasmid as a matrix. A third fragment was amplified using GA1 genome as a matrix with chimeric primers designed to obtain a *srf* promoter amplicon flanked by 20-bp connectors in 5′ and 3′ containing homologies to the upper and lower *amyE* fragments, respectively. All three fragments were joined with a second PCR race to obtain the final cassette. *B. velezensis* GA1 transformation was performed after modification from the protocol developed by Jarmer et al. (67). Briefly, one colony was inoculated into LB liquid medium at 37°C (160 rpm) during 6 h, and cells were washed two times with peptone water. A total of 1 μg of the recombinant cassette was added to the GA1 cell suspension adjusted to an OD_600_ of 0.01 into MMG liquid medium (19 g liter^−1^ K_2_HPO_4_ anhydrous, 6 g liter^−1^ KH_2_PO_4_, 1 g liter^−1^ Na_3_ citrate anhydrous, 0.2 g liter^−1^ MgSO_4_ 7H_2_O, 2 g liter^−1^ Na_2_SO4, 50 μM FeCl_3_ [sterilized by filtration at 0.22 μm], 2 μM MnSO_4_, 8 g liter^−1^ glucose, and 2 g liter^−1^ l-glutamic acid; pH 7.0). Cells were incubated at 37°C with shaking, and colonies that integrated the cassette by a double crossing over event were selected on an LB plate supplemented with chloramphenicol. Proper integration of the *cat*-*gfp* locus was verified by PCR. Knockout mutant strains were constructed by gene replacement by homologous recombination. A cassette containing a chloramphenicol resistance gene flanked by 1 kb of the upstream region and 1 kb of the downstream region of the targeted gene was constructed by a three partner overlap PCR. This recombination cassette was also introduced in *B. velezensis* GA1 by inducing natural competence as described above (67). A double homologous recombination event was selected by chloramphenicol resistance. Deletion was confirmed by PCR analysis with the corresponding upstream and downstream primers.

**TABLE 2:** Strains used in this study

Strain by species	Characteristic(s)	Source
Bacillus velezensis		
GA1	Wild-type strain	84
GA1 Psrf_gfp	*amyE*::Psrf*_gfp+chl*; Chl+	This study
GA1 Δ*srfAA*	Δ*srfAA*::*chl*; Chl+	This study
GA1 Δ*codY*	Δ*codY*::*chl*; Chl+	This study
S499	Wild-type strain	15
FZB42	Wild-type strain	13
QST713	Wild-type strain	85
Bacillus amyloliquefaciens		
DSM 7	Wild-type strain	ATCC
Bacillus subtilis		
ATCC 21332	Wild-type strain	ATCC
Bacillus pumilus		
QST 2808	Wild-type strain	86
Escherichia coli		
dh5α	Wild-type strain	CGSC
dh5α pGEM-T Easy *amyE*	pGEM-T Easy *amyE*; Amp+	This study
dh5α pGEM-T Easy *amyEup-cat-gfp-amyEdw*	pGEMT-T Easy *amyEup-cat-gfp-amyEdw*; Amp+ Chl+	This study
dh5α pGFP_Star	pGFP-Star; Chl+	This study

### Fluorescence measurement.

Fluorescence accumulation was evaluated with the channel FL1 of a BD Accuri C6 flow cytometer (Biosciences) with the following parameters: 20,000 events, medium flow rate (35 μl min^−1^), and a forward scatter (FSC) threshold of 20,000.

### Genome sequencing.

The GA1 genome sequence was reconstructed using a combined approach of two sequencing technologies which generated short paired-end reads and long reads. The resulted sequences were then used for hybrid assembly. More precisely, genomic DNA was extracted and purified from *B. velezensis* GA1 using the GeneJET genomic DNA purification kit (ThermoFisher Scientific). The first half of extracted DNA was sent to the GIGA sequencing facility (Liège, Belgium) and used as the DNA template for Illumina MiSeq sequencing after being prepared using the Nextera library kit (Illumina). The sequencing run generated 150-bp paired-end reads, which were trimmed and corrected using an in-house python script and SPAdes 3.14 (68) before assembly. The second half of the extracted DNA was used to generate long reads with a MinION Oxford Nanopore platform. A DNA library was constructed using the rapid sequencing kit (SQK-RAD0004; Oxford Nanopore). Adapters were trimmed from generated reads with Porechop software (https://github.com/rrwick/Porechop). Trimmed reads were then filtered by size (>500) and Q-score (>10) using NanoFilt implemented in NanoPack (69). Finally, the hybrid assembly was performed using the hybridSPAdes algorithm implemented in SPAdes 3.14 (70).

### Transcriptome library preparation and sequencing.

RNA extraction was performed for each sample using the NucleoSpin RNA kit (Macherey-Nagel). Total RNAs were quantified using a Nanodrop instrument (ThermoFisher). For sequencing, all samples were sent to the GIGA genomics platform in Liège, Belgium. Genome quality was assessed using the RNA 6000 Nano Chip kit on a 2100 bioanalyzer (Agilent). cDNA libraries were prepared by employing the universal prokaryotic transcriptome sequencing (RNA-seq), prokaryotic AnyDeplete kit (Nugen) according to the manufacturer’s instructions. cDNA libraries were quantified and normalized by using the Kapa SYBR fast mastermix (Sigma-Aldrich) with P5-P7 Illumina primers according to the manufacturer’s instructions. Prepared libraries were sequenced on a NextSeq 550 device (Illumina) by using the following parameters: paired end, 80 cycles read 1, 8 cycles index, and 80 cycles read 2.

### RNA-seq data analysis.

The raw RNA-seq reads were trimmed using Trimmomatic v0.39 (71). We performed a quality-control step on the trimmed reads using FastQC v0.11.8 (Babraham Bioinformatics). Trimmed reads were mapped to the GA1 reference genome (see section “Genome sequencing” for accession numbers) using BWA-MEM v0.7.17 (72) with the following settings: mem -k 50 -B 40 -v 1. At least 95.4% of reads uniquely mapped to the annotated reference genome. SAMtools v1.9 (73) was used to generate the BAM files and their indices. To calculate the read counts, the python-based tool HTSeq v0.9 (74) was employed with the following parameters: htseq-count -q -s no -f. The Cufflinks function cuffnorm (75) was used to generate the fragments per kilobase of transcript per million mapped reads (FPKM) tables using the following settings: –compatible-hits-norm –library-norm-method classic-fpkm. Genes with low reads counts (<25) were removed before further analysis. A differential expression analysis was conducted according to the DESeq2 pipeline (76) with cutoff parameters as follows: *P* value of <0.05 and log_2_ fold change of >1.5.

### Motility and biofilm assays.

Swarming motility assays were performed according to Molinatto et al. (77). The diameter of the bacterial swarming pattern was measured 48 h after inoculation on REM soft agar plates (0.8% agar) supplemented or not with 0.1% HGLM. Quantification of the total biofilm was performed by crystal violet staining. A strain of interest was inoculated at a final OD_600_ of 0.1 in a 96-well microplate containing 200 μl of REM supplemented or not with 0.1% HGLM. The plate was incubated at 30°C during 24 h without shaking. Medium and planctonic cells were discarded and wells were washed with phosphate-buffered saline (PBS). The biofilm pellicle was stained with 0.1% crystal violet during 10 min and washed with PBS. The stained biofilm was dissolved with 30% acetic acid. Absorbance was measured at 595 nm.

### Plant growth conditions and root colonization assays.

For sterilization, tomato seeds were first immersed in a 70% ethanol solution for 2 minutes, transferred in a 20% bleach solution under shaking conditions for 20 minutes, and rinsed three times with sterile water. Sterilized tomato seeds were pregerminated on solid Hoagland medium at 22°C under a 16 h/8 h night/day cycle. After 4 days, 5 μl of cultures containing the strain of interest and calibrated at an OD_600_ of 1 was deposited on the root top. After 1 and 3 days of colonization, roots were harvested, deposited separately in a peptone water solution supplemented with 0.1% of Tween, and vortexed vigorously to tear off the bacterial cells from the roots. Several dilutions were plated on LB media to evaluate the level of colonization. Measurements of surfactin production by GA1 cells colonizing roots were performed on 1- by 1- by 0.7-cm pieces of gelified medium containing roots based on the assumption that the produced lipopeptide diffused to a maximal distance of 5 mm from each part of the root and is uniformly distributed over the surface as we previously observed via imaging MS (78). A 10-fold concentration factor was applied to estimate concentrations around the root surface in order to take into account diffusion constraints in a solid matrix. Surfactin was quantified by UPLC-MS as described below.

### Plant cell wall extraction.

Tobacco seeds were sterilized as described above for tomato seeds and deposited on Hoagland plates at 22°C for 1 week for a successful germination process. Each plantlet was then transferred in a seedholder filled with soft agar and put in Araponics boxes containing the nutritive solution described above. Cell wall extraction was performed on 6-week-old plants grown at 22°C with a 16 h/8 h day/night cycle. Roots were harvested, lyophilized, and reduced to powder using a Retsch MM400 grinder. A total of 500 mg of powder was resuspended in 40 ml of ethanol 80% at 90°C for 20 min. The insoluble cell wall fraction was recovered by centrifugation, and the pellet obtained was washed once with water to obtain the alcoholic insoluble residue (AIR) used for fractionation. The AIR was freeze-dried before use in a fractionation protocol. The sequential extraction of root cell walls was performed using a protocol derived from Carpita (79) and Silva et al. (80). Dry AIR was resuspended in 40 ml of water and incubated at 100°C for 20 min. The supernatant was recovered after centrifugation as a soluble pectic fraction (cPEC).

### Monosaccharide composition analysis using HPAEC-PAD.

Before the monosaccharide composition analysis, the cPec fraction was dialyzed for 24 h against a large volume of water and freeze-dried. A total of 2 mg of dried fraction material was hydrolyzed in 1 ml of 2 M trifluoroacetic acid (TFA) at 121°C for 90 min. TFA was evaporated under nitrogen gas flux, and the hydrolyzed dried residue was resuspended in 1 ml water, filtered on a 0.2-μm cartridge, and stored in vials at 20° before high-performance anion exchange chromatography with pulsed amperometric detection (HPAEC-PAD). HPAEC-PAD was used for neutral and acidic monosaccharide composition analysis using a DX-500 system (Dionex Corporation) equipped with a Carbopac PA-1 analytical column (4 mm by 250 mm). The elution was performed with a flow rate of 1 ml min^−1^ in a gradient mode. The gradient for neutral sugars (eluent A, deionized water; eluent B, 160 mM NaOH; and eluent C, 200 mM NaOH) was 10% B for 25 min, 100% B for 10 min, and finally an equilibration step with 10% B (15 min). The gradient for uronic acid (eluent A, 160 mM NaOH; and eluent B, 160 mM NaOH + 600 mM AcONa) was 0% B for 5 minutes, 30 minutes of linear gradient from 0% to 100% B, 100% B for 5 minutes, and finally an equilibration step with 0% B (10 minutes). Detection was performed with a pulsed amperometric ED50 detector (Dionex Corporation). A total of 20 ml of the sample was injected with an autosampler. Each carbohydrate concentration was determined after integration of the respective areas (Chromeleon management system; Dionex) and comparison with standard curves.

### LC-MS analyses.

The detection of metabolites and quantification was performed by LC-MS. A total of 10 μl of samples was used for UPLC-MS with UPLC (Acquity H-class; Waters) coupled to a single quadrupole mass spectrometer (SQD mass analyzer; Waters) using a C_18_ column (Acquity UPLC BEH C_18_; 2.1 mm by 50 mm, 1.7 μm). Elution was performed at 40°C with a constant flow rate of 0.6 ml/min using a gradient of acetonitrile (solvent B) and water (solvent A) that were both acidified with 0.1% formic acid as follows: starting at 15% B during 2 min, solvent B was then raised from 15% to 95% in 5 min and maintained at 95% up to 9.5 min before going back to initial conditions at 9.8 min during 3 minutes before the next injection if needed. Compounds were detected in electrospray positive ion mode by setting SQD parameters as follows: source temperature, 130°C; desolvation temperature, 400°C; and nitrogen flow, 1,000 liter h^−1^ with mass range from *m/z* of 800 to 1,550. Surfactins were quantified based on their retention times and masses compared with commercial standards (98% purity; Lipofabrik).

### Induction of systemic resistance and ROS measurements.

ISR assays were performed as described previously (39) on 4-week-old tobacco plants cultivated under hydroponic conditions using the Hoagland solution as a nutrient base. Plants were treated with pure surfactin at the root level and infected on leaves by applying a spore suspension of the phytopathogen *Botrytis cinerea* prepared as detailed previously (39). Spreading lesions occurred starting from 48 h postinfection, and the diameter size was measured 2 days later. Five plants were used per treatment, and experiments were repeated independently at least twice. For the determination of cytoplasmic ROS stimulation, a fluorescent probe (dichloro-dihydro-fluorescein diacetate [DCFH-DA]) was used. Plants used in this experiment were grown on Hoagland medium for 2 weeks as described above. Experiments were performed on nine samples per treatment, with each containing three root segments (approximately 100 mg fresh weight [FW]) collected from different plants (*n* = 9). Roots were treated with 50 μM DCFH-DA for 10 minutes, rinsed with PBS upon removing the probe, and finally treated. All operations were conducted in a 96-well black microplate. Fluorescence measurements were performed on a Spark (Tecan) microplate reader (excitation, 485 nm; emission, 535 nm) with readings every 10 minutes. Stimulation of apoplastic hydrogen peroxide production in root cells was measured via chemiluminescence (ferricyanide-catalyzed oxidation of luminol). Means and standard deviations were calculated from measurements performed on three samples per treatment, with each containing three root segments (approximatively 100 mg FW) collected from different plants. Extracellular ROS in tomato roots was conducted according to Bisceglia et al. (81) with minor changes. Namely, instead of leaf discs, tomato roots, with three segments (approximatively 100 mg FW from the same plant) per sample, were used. Plants were grown for 2 weeks on Hoagland medium, and chemiluminescence was measured in a Tecan Spark plate reader.

### ITC analysis.

ITC analyses were performed with a VP-ITC microcalorimeter (Microcal). The calorimeter cell (volume of 1.4565 ml) was filled with a 10 μM (below the CMC concentration) surfactin solution in buffer (10 mM Tris, 150 mM NaCl, and 1 mM EDTA at pH 8.5). The syringe was filled with a suspension of large unilamellar vesicles (LUV) at a lipid concentration of 5 mM. A series of 10-μl injections was performed at constant time intervals (6 min) at 25°C. The solution in the titration cell was stirred at 305 rpm. Prior to each analysis, all solutions were degassed using a sonicator bath. The heats of dilution of vesicles were determined by injecting vesicles in buffer and subtracted from the heats determined in the experiments. Data were processed by software Origin 7 (Originlab) using the cumulative model described by Heerklotz and Seelig (82). All measurements were repeated at least three times with two different vesicle preparations.

### Leakage assays.

Membrane permeabilization was followed as described by Van Bambeke et al. (83). Release of 8-hydroxypyrene-1,3,6 trisulfonic acid (HTPS) coentrapped with and quenched by p-xylene-bis-pyridinium bromide (DPX) from liposomes can be monitored by the fluorescence increase upon dilution following their leakage from the vesicles. Surfactin C_12_ or surfactin C_14_ was added from a stock solution in dimethyl sulfoxide (DMSO), and fluorescence intensities were recorded immediately. The percentage of HPTS released was defined as [(*Ft* − *Fcontr*)/(*Ftot* − *Fcontr*)]/100, where *Ft* is the fluorescence signal measured after 15 min in the presence of surfactin C_12_ or surfactin C_14_, *Fcontr* is the fluorescence signal measured at the same time for control liposomes, and *Ftot* is the total fluorescence signal obtained after complete disruption of the liposomes by 0.05% Triton X-100. All fluorescence determinations were performed at room temperature on a LS-50B fluorescence spectrophotometer (Perkin-Elmer Ltd.) using λexc of 450 nm and a λem of 512 nm.

### Statistical analyses.

All statistical analyses were performed on GraphPad Prism. Before each statistical analysis, variance homoscedasticity was verified by using a Brown-Forsythe test. Analysis of variance (ANOVA) was used for multiple comparisons, and significant differences were indicated by different letters. Statistical differences between means were evaluated by two-tailed Student’s *t* test. The number of biological replicates used for each experiment are indicated in the corresponding figure legend. *P* values are indicated in the figure legends.

### Data availability.

The RNA-seq data sets produced for this study are deposited at https://www.ebi.ac.uk/ena/ under the project reference PRJEB39762. All other data sets analyzed for this study are included in the supplemental files. The Genome Resulting assembly of the GA1 strain was deposited in the GenBank database under the accession numbers CP046386 and CP046387.
