# Supplementary figures and images for: Surfactin Stimulated by Pectin Molecular Patterns and Root Exudates Acts as a Key Driver of the Bacillus-Plant Mutualistic Interaction
Source: mBio. 2021 Nov 2;12(6):e01774-21. doi: 10.1128/mBio.01774-21 (PMC8561381; doi:10.1128/mBio.01774-21)

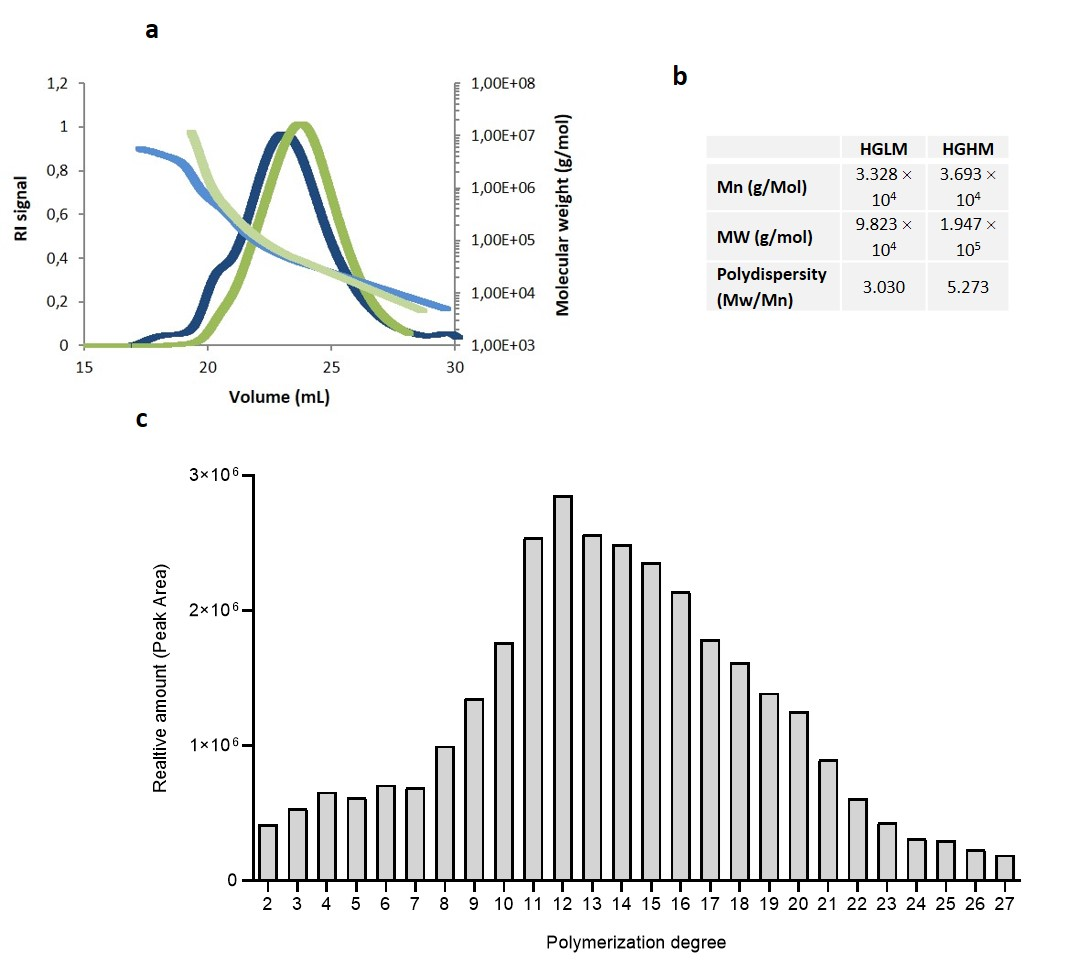

Supplement: FIG S1 [file mbio.01774-21-sf001.tif]

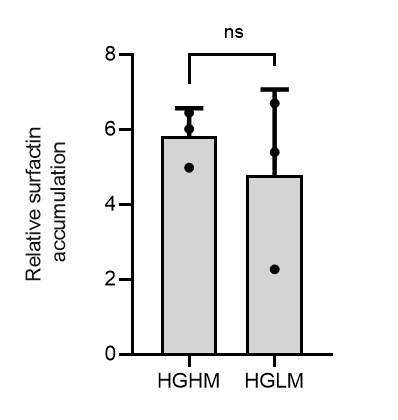

Supplement: FIG S2 [file mbio.01774-21-sf002.tif]

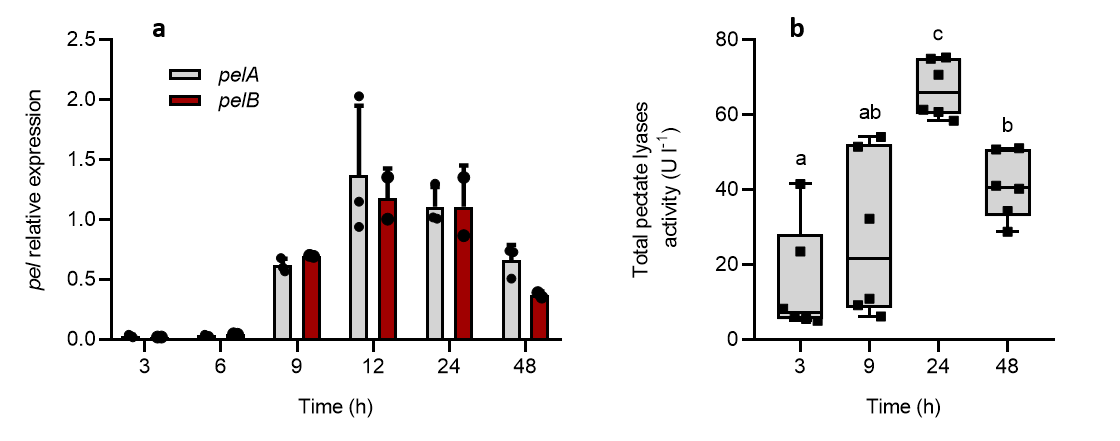

Supplement: FIG S3 [file mbio.01774-21-sf003.tif]

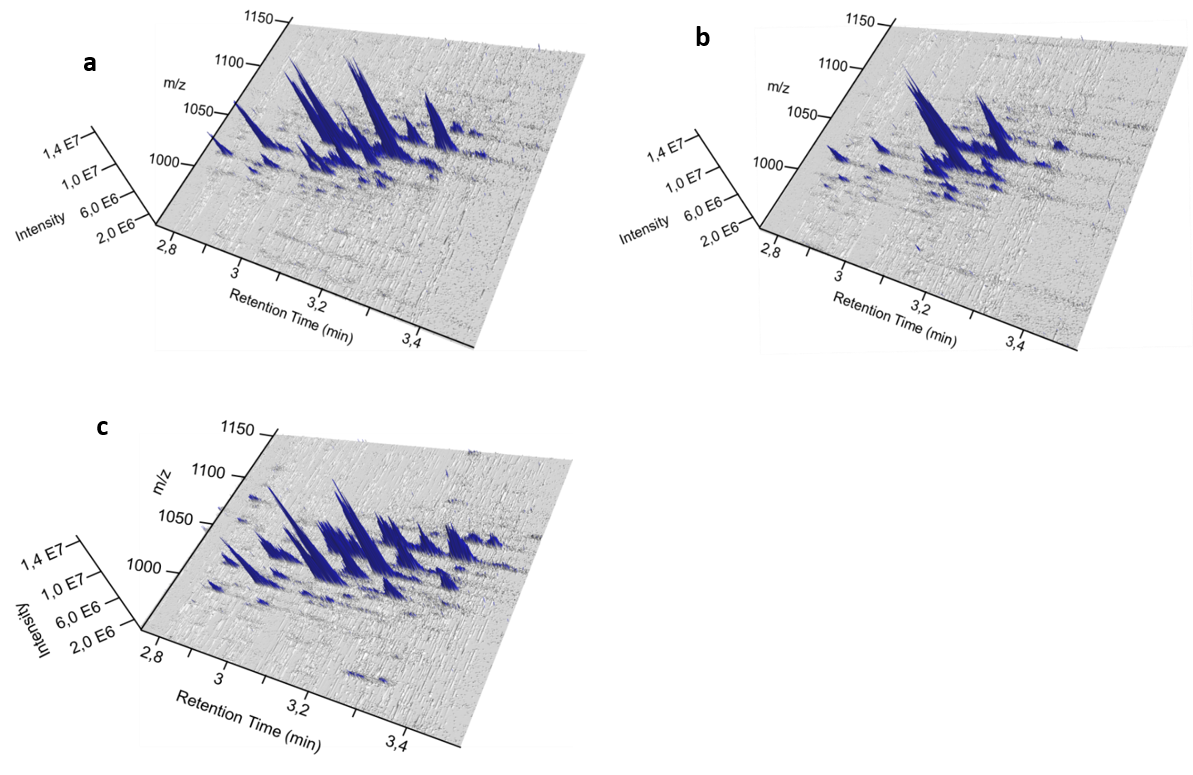

Supplement: FIG S4 [file mbio.01774-21-sf004.tif]

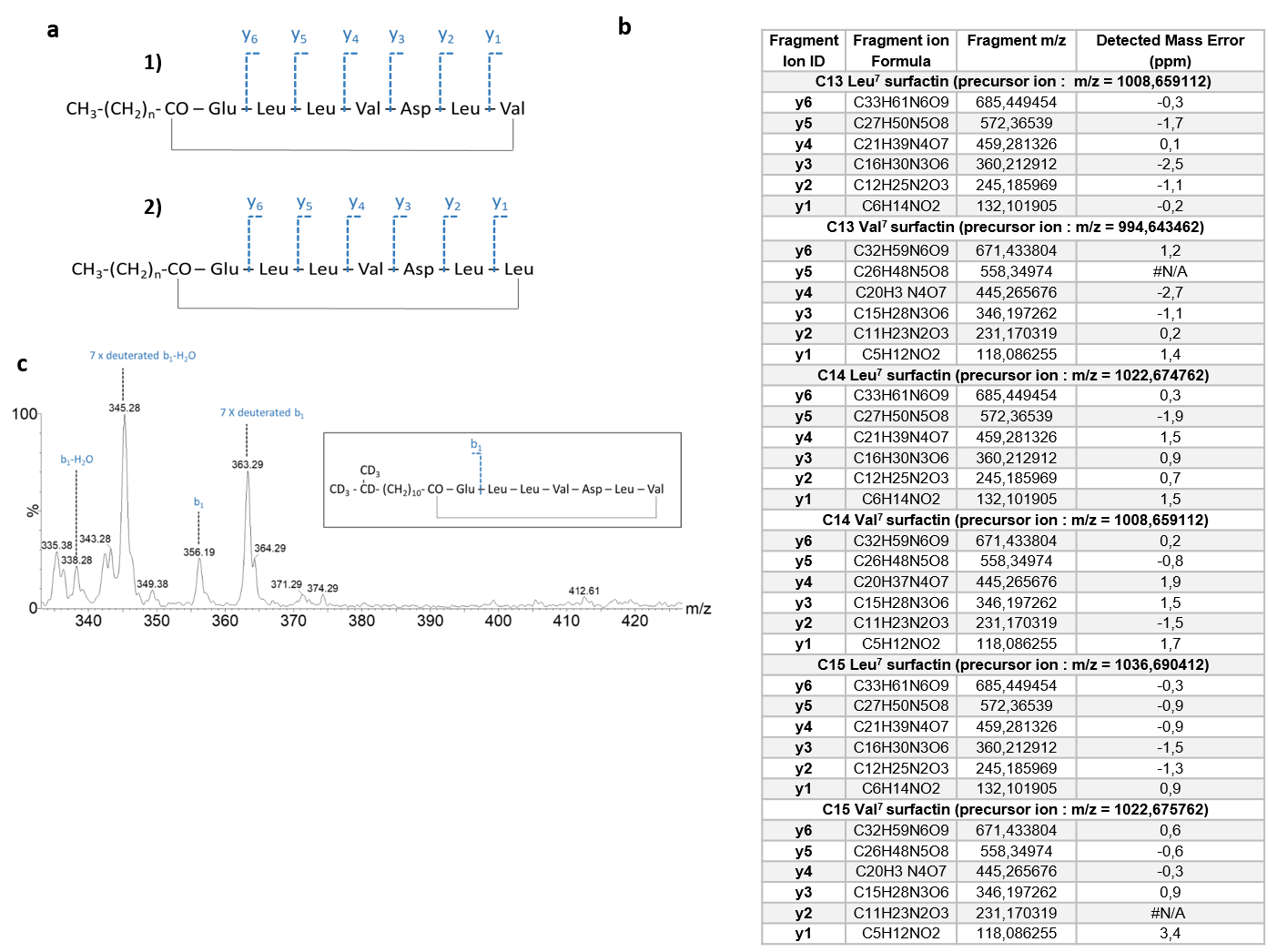

Supplement: FIG S5 [file mbio.01774-21-sf005.tif]

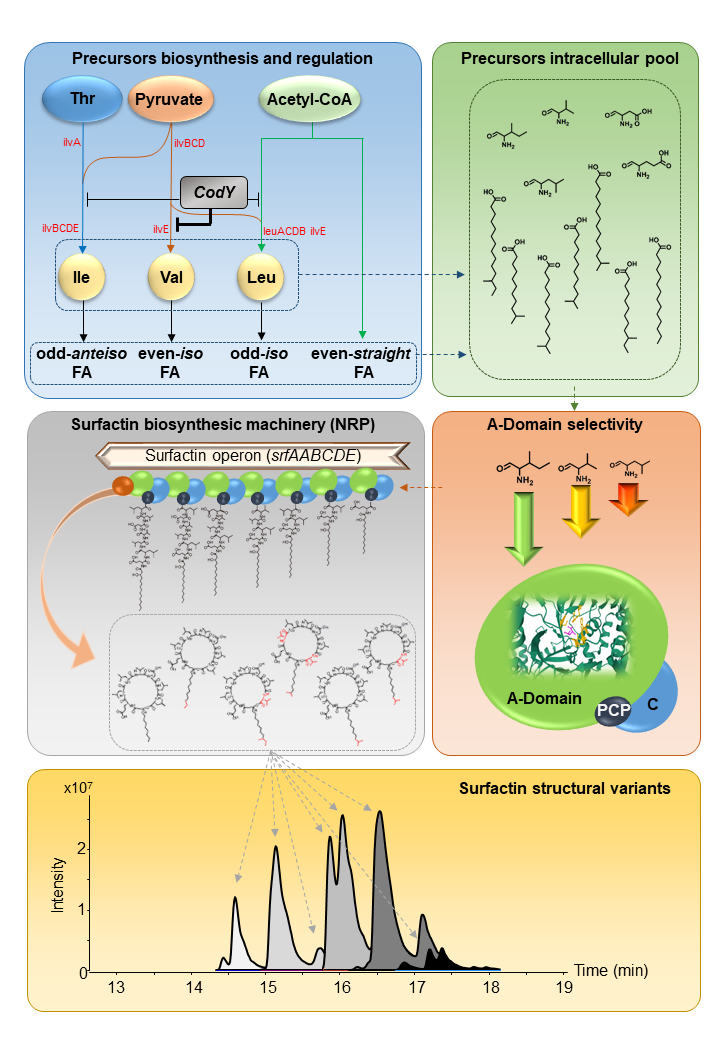

Supplement: FIG S6 [file mbio.01774-21-sf006.tif]

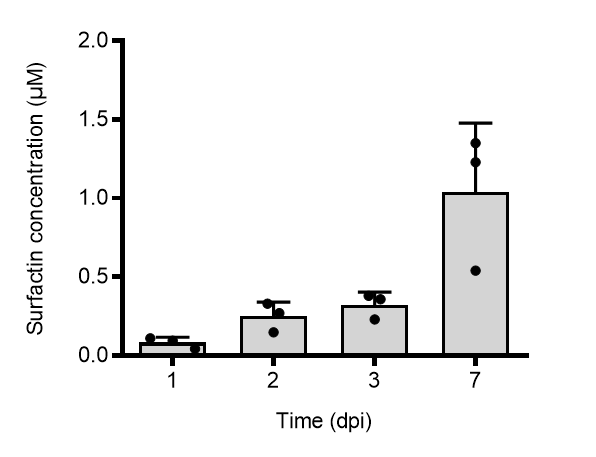

Supplement: FIG S7 [file mbio.01774-21-sf007.tif]
